# New York State Climate Impacts Assessment Chapter 07: Human Health and Safety

**DOI:** 10.1111/nyas.15244

**Published:** 2024-12-09

**Authors:** Janice Barnes, Perry Sheffield, Nathan Graber, Sonal Jessel, Kevin Lanza, Vijay S. Limaye, Faustenia Morrow, Anjali Sauthoff, Michael Schmeltz, Shavonne Smith, Amanda Stevens

**Affiliations:** ^1^ Climate Adaptation Partners New York New York USA; ^2^ Department of Environmental Medicine and Public Health Icahn School of Medicine at Mount Sinai New York New York USA; ^3^ Pediatrics, Albany Medical Center Albany New York USA; ^4^ WE ACT for Environmental Justice New York New York USA; ^5^ Environmental and Occupational Health Sciences The University of Texas Health Science Center at Houston School of Public Health Austin Texas USA; ^6^ Natural Resources Defense Council New York New York USA; ^7^ Monarch of Infinite Possibilities Buffalo New York USA; ^8^ Westchester County Climate Crisis Task Force and Independent Environmental Health Consultant Pleasantville New York USA; ^9^ Department of Public Health California State University at East Bay Hayward California USA; ^10^ Environmental Department Shinnecock Indian Nation Southampton New York USA; ^11^ New York State Energy Research and Development Authority Albany New York USA

**Keywords:** adaptation, climate change, human health, impacts, New York State, public health, resilience, vulnerability

## Abstract

New Yorkers face a multitude of health and safety risks that are exacerbated by a changing climate. These risks include direct impacts from extreme weather events and other climate hazards, as well as indirect impacts occurring through a chain of interactions. Physical safety, physical health, and mental health are all part of the equation—as are the many nonclimate factors that interact with climate change to influence health outcomes. This chapter provides an updated assessment of all these topics at the intersection of climate change, public health and safety, and equity in the state of New York. Key findings are presented below.

## 1 TECHNICAL WORKGROUP KEY FINDINGS

New Yorkers face a multitude of health and safety risks that are exacerbated by a changing climate. These risks include direct impacts from extreme weather events and other climate hazards, as well as indirect impacts occurring through a chain of interactions. Physical safety, physical health, and mental health are all part of the equation—as are the many nonclimate factors that interact with climate change to influence health outcomes. This chapter provides an updated assessment of all these topics at the intersection of climate change, public health and safety, and equity in the state of New York. Key findings are presented below.

**Key Finding 1: Climate change poses escalating health and safety risks for New Yorkers from heat, heavy rainfall, flooding, and air quality changes, combined with nonclimate stressors**. However, public health is not a key focus of most of the state's planning initiatives to address climate change. New York agencies and organizations can consider ways to reduce impacts by raising and tracking health risk awareness, offering near‐term coping resources, participating in the development of hazard mitigation strategies to incorporate health outcomes, and framing adaptation strategies to include training (for health professionals, the general public, and high‐risk populations) and health‐indicator monitoring.

**Key Finding 2: Climate change–related impacts on mental health and well‐being are pronounced**. Heat, flooding, extreme storms, and other climate‐related events have documented, detrimental effects on mental health—especially for children and youth, older adults, people with pre‐existing mental health concerns, and those with limited access to mental health resources (such as many Indigenous and rural communities and unhoused individuals). Raising awareness of climate‐related mental health concerns can give New York health service providers the information they need to engage with patients on the topic and encourage actions to reduce exposure to climate hazards and their associated mental stresses, provide guidance on adaptations to deal with growing health risks, assuage anxieties, and offer grounded hope as well as applied solutions.

**Key Finding 3: Discriminatory systems and policies amplify climate change–related health risks for New Yorkers**. Structural racism—a particularly entrenched form of discrimination—refers to the many ways in which this discrimination against people of color and Indigenous Peoples exists within systems and policies and how this affects access to and the distribution of resources. Such systemic forms of discrimination are embedded across housing, employment, health care, and other systems and result in people of color and low‐income and Indigenous Peoples having less adequate access to care, worse health outcomes, and greater climate exposures than other populations, making them more vulnerable to climate change impacts. Addressing climate change–related health impacts means also addressing systemic discriminatory policies and practices—particularly structural racism—and their long‐term negative health outcomes.

**Key Finding 4**: **A collaborative, health‐centric, “whole of community” approach is essential for addressing the urgency and broad impacts of climate change**. Municipal and county health departments are at the front lines for climate response. However, because climate impacts vary greatly between and within communities, adaptation strategy development would also benefit from including families, businesses, community‐based organizations, and institutions who are affected. Efforts to engage local health departments in municipal and state planning for climate change have begun, and drawing diverse community members and organizations into the process can be a valuable next step.

**Key Finding 5: Public health efforts to address climate change must be sustained to be effective**. Public health agencies face competing challenges, and their climate‐related efforts are susceptible to suspension when other health crises occur. If New York wants to sustain public health agencies’ focus on climate change, local health departments will need discrete leadership, funding, and staffing. Given the projected rise in disease exposure and climate‐related increases in pandemics, this represents a critical step in preparing health systems, governments, and individuals.

BOX 1 **Developments since the 2011 ClimAID assessment**

The 2011 ClimAID assessment's public health chapter anticipated an increase in heat‐related deaths, diverse consequences from more intense rainfall and flooding events, worsening air quality and related respiratory health impacts, changing patterns of vector‐borne and other infectious diseases, and other health impacts. This updated chapter focuses on similar issues. It also provides examples of relevant social determinants of health, structural inequities, and compounding and cascading effects. Today, health practitioners more fully recognize that social determinants or “conditions in which people are born, grow, work, live, and age”^1^ affect public health. Although rooted in the history of public health, emphasis on social determinants of health increased substantially in the decade following the 2012 World Health Organization report *Social Determinants of Health: Report by the Secretariat*.^2^ In addition, there is more recognition of the social, environmental, and structural disadvantages and stressors (such as poor housing quality or lack of access to open spaces, nutritious food, and health care) that exacerbate climate impacts and contribute to poor health outcomes.^3^, ^4^ There is also greater and evolving awareness and understanding that climate impacts can occur simultaneously and interact with one another, compounding and cascading across timescales, sectors, and regions.

While various consortia, networks, and policy action agendas have increased focus on integrating climate considerations into health practices around the world since 2011,^5^, ^6^, ^7^, ^8^ neither New York State nor the United States has fully integrated public health into climate change preparedness and recovery efforts. Climate considerations are not typically part of patient monitoring in health practices,^9^ though the situation may be changing.^10^, ^11^, ^12^, ^13^ For example, New York is increasing climate and health data tracking and related research as well as improving characterization via the New York State Environmental Public Health Tracker and the Climate and Health Hub.^14^, ^15^ Despite this progress, public health remains on the perimeter of climate change hazard mitigation and adaptation. As a result, public health practitioners must continuously advocate for its inclusion in climate change adaptation efforts. Without public health considerations, ingrained disparities that amplify population vulnerabilities and inequities remain less visible.^16^

## 2 INTRODUCTION AND BACKGROUND

Climate change poses many threats to health and safety across New York State. For example, more severe heat waves can lead to more heat‐related illnesses, such as heat exhaustion or heat stroke. Additionally, warmer temperatures can lead to adverse birth outcomes, kidney disease, and negative impacts for those with respiratory illnesses and cardiovascular disease. Warmer temperatures can also create prime conditions for ticks to expand their range, potentially increasing the risk of illnesses such as Lyme disease. Flooding can contaminate water supplies and spread waterborne illnesses. Rising sea levels can drive displacement, affecting social ties and support systems. Extreme weather events, such as storms and floods, can cause physical injuries and mental health effects, including post‐traumatic stress disorder (PTSD). These are just some of the possible direct effects of climate change on health in New York State, and they are not expected to be isolated incidents; communities can be affected by multiple impacts, compounding the risk of harm.

Health and safety in New York will also be affected by indirect impacts. For example, extreme weather can cause power outages and damage buildings, which not only leaves people vulnerable at home but also threatens access to emergency or medical care and even the ability of medical facilities and equipment to function. Moreover, many of the interconnected systems and services that are critical to health—including medical care, buildings and energy, food and water, transportation, and natural environments—are vulnerable to climate impacts.

This chapter provides a detailed review of observed and projected impacts of climate change on human health and safety, both direct and indirect. It also explores how issues of community vulnerability, equity, and environmental justice affect health and safety as the climate changes; a range of cascading, compounding, and cross‐cutting issues; and opportunities to adapt and build resilience. Specifically:

- **Section** **3** provides a comprehensive review of the current and projected health and safety impacts of climate hazards such as extreme temperature, storms and floods, and sea level rise. This section also discusses specific adaptation strategies that individuals and organizations could implement or are already implementing to cope with the uncertainties of a changing climate.
- **Section** **4** discusses climate justice considerations and disproportionate impacts on vulnerable communities, including Indigenous Peoples, people of various ages and occupations, people living with pre‐existing conditions and housing challenges, and others. It also discusses the many nonclimate challenges that intersect with climate change and increase risks to health and safety for all New Yorkers.
- **Section** **5** broadens the discussion to include compounding, emerging, and cross‐cutting issues and how health and safety connect and interact with other sectors.
- **Section** **6** concludes the chapter with some thoughts on opportunities and emerging climate health research needs to help New York State better understand and prepare for climate impacts on the health sector.
- The **Traceable Accounts** appendix examines each key finding in depth. It provides citations that support each assertion, and it presents the authors’ assessment of confidence in each finding.
- **Case studies** highlight key climate impacts affecting health and safety as well as adaptation and resilience strategies that can minimize their effects on communities. These case studies are not included in the chapter proper but are available through links provided in the chapter.

### 2.1 Sector scope and context

This chapter focuses on the impacts of realized and projected climate changes on human health and safety. The concepts of health and safety are inexorably intertwined. Here, health refers to physical and mental well‐being, while safety refers to the ability to conduct life without risk of harm. Safety addresses emergency preparedness and response as well as occupational safety via environmental management.

Many health and safety concerns bear some relationship to climate conditions. The U.S. Global Change Research Program has grouped these concerns into the following categories^17^:

- Temperature‐related death and illness (both heat‐ and cold‐related).
- Air quality, including air pollution, aeroallergens, and wildfire smoke.
- Extreme events such as large storms and floods, which can cause death and direct physical harm as well as serious residual effects (e.g., mold in homes).
- Vector‐borne diseases, including those carried by mosquitoes, ticks, and fleas.
- Water‐related illness resulting from exposure to water, fish, or shellfish contaminated with harmful microorganisms or toxins.
- Food safety, nutrition, and distribution, including foodborne illness and access to food.
- Mental health and well‐being, which can be affected by disasters, temperature, and the threat of climate change itself.

Figure 7‐1 illustrates the general mechanism through which climate hazards influence health outcomes. Borrowing from the field of chemical risk assessment, scientists often identify exposure pathways when describing the main routes by which climate hazards affect human health. Climate hazards (such as increasing temperatures, extreme precipitation, sea level rise, and extreme events) directly impact physical settings (environmental and institutional context) as well as populations (social and behavioral context). These in turn influence exposure pathways (if and how people are exposed), which include extreme heat, poor air quality, reduced food and water quality, changes in infectious agents, and population displacement.

**FIGURE 7‐1:**
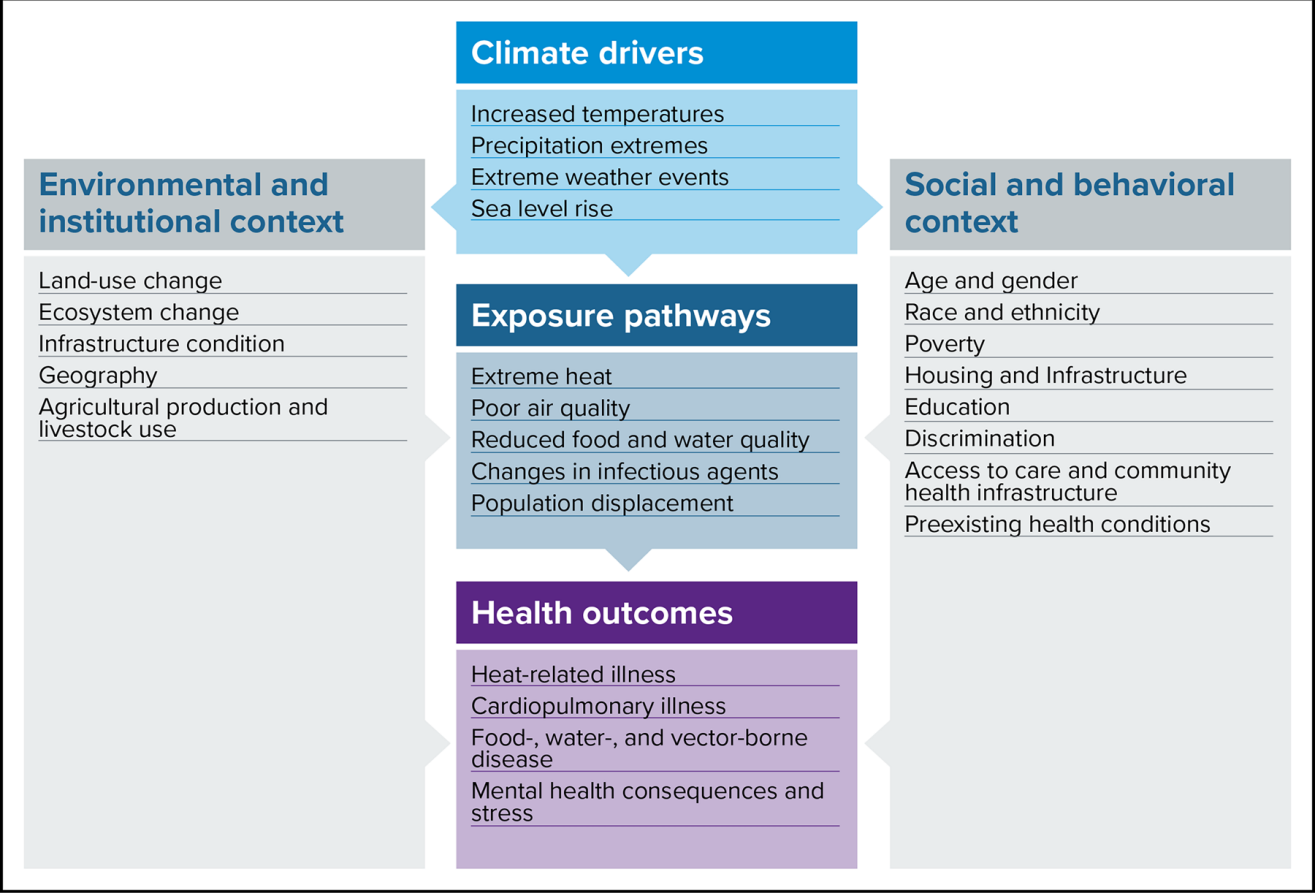
Climate change and health. This figure represents the U.S. Global Change Research Program's framing of climate change and health. While the concepts apply to this assessment, specific terminology (e.g., drivers/hazards, outcomes/impacts) may vary. Figure from U.S. Global Change Research Program (2016).^17^

Figure 7‐1 also shows how the environmental and institutional or social and behavioral context can drive health outcomes that are also influenced by exposure to a climate hazard. Health impacts can be further amplified by interrelationships with infrastructure (e.g., wastewater sanitation, transportation options) and energy, water, and food sources.^18^ For example, rising temperatures in New York State affect the availability of certain fish that coastal Indigenous Peoples rely on for sustenance. These interactions reduce the availability and quality of food sources, affecting health (refer to the Water Resources and Society and Economy chapters). The interrelationships between climate hazards, physical and social contexts, and exposure pathways contribute to health outcomes.

Every New Yorker faces health and safety impacts associated with climate change. However, some people face higher risks than others because of differences in vulnerability. The Assessment Introduction explains three key components of vulnerability: *exposure* (contact with a hazard), *sensitivity* to the hazards to which one is exposed, and *adaptive capacity* (having the ability and resources to respond and adjust to hazards).^17^ These elements of vulnerability vary across individuals and populations. Figure 7‐2 provides an example of how vulnerabilities in all three categories can be identified for a particular hazard and exposure pathway—in this case, extreme heat. The diagram in Figure 7‐2 also identifies external drivers and adaptation options.

**FIGURE 7‐2:**
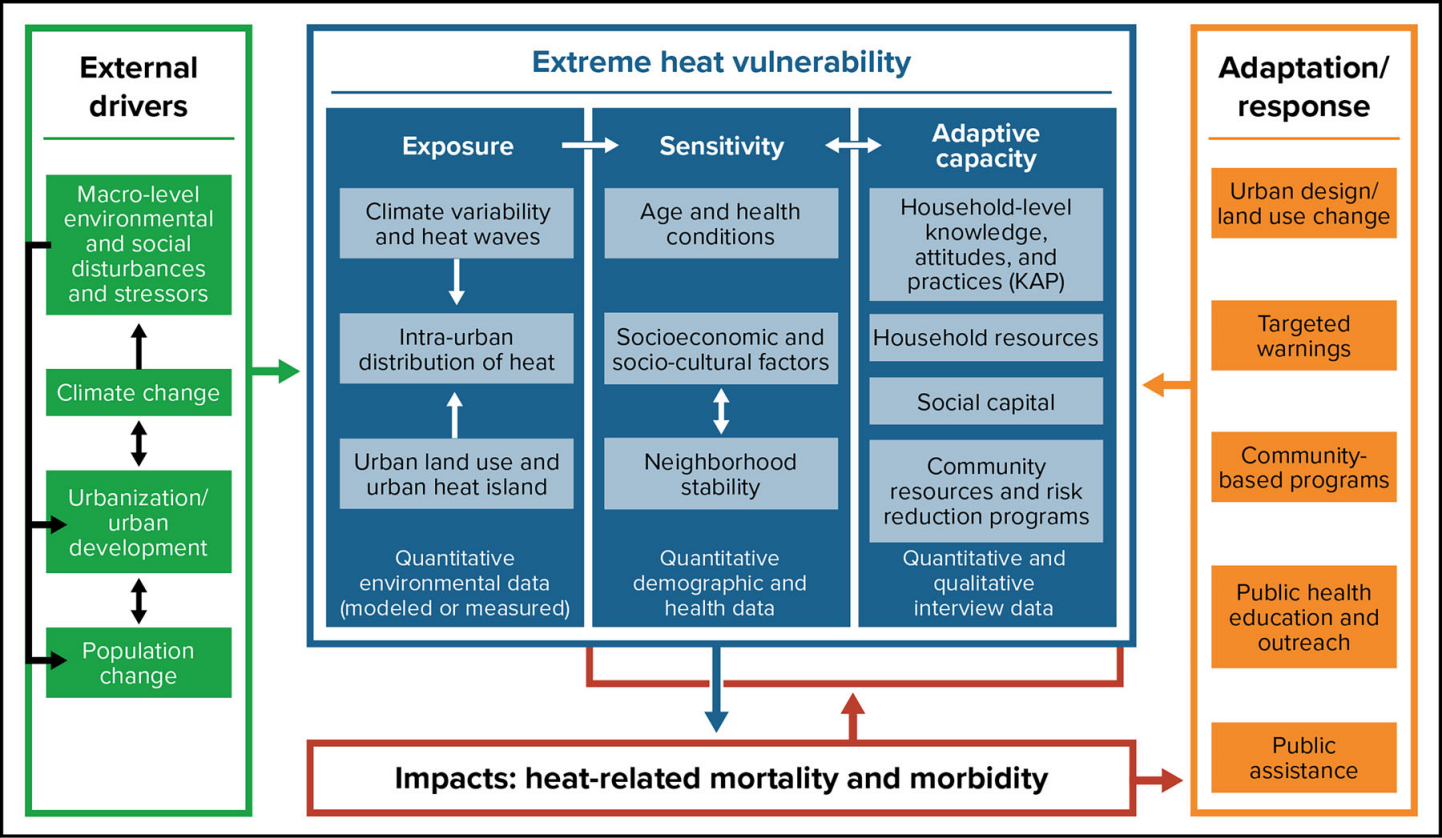
Extreme heat vulnerability analysis framework. Figure from Wilhelmi and Hayden.^19^

Sections 2.4 and 2.5 introduce some of the groups that face elevated risks due to social, environmental, economic, and structural factors—such as poor housing quality or lack of access to open spaces, nutritious food, and health care—that can exacerbate poor health outcomes.^3^, ^4^ Section 4 discusses these vulnerabilities in more depth, along with opportunities to address them and reduce longstanding disparities.

This assessment acknowledges that climate change acts within dynamic social, economic, demographic, and environmental contexts, creating interacting hazards and risks. Effective management of climate impacts requires an understanding of the systemic interconnections that affect human, environmental, and animal health—a paradigm called One Health.^20^ Recognition of One Health is fundamental to a deeper understanding of socio‐eco‐biological interactions, which ultimately affect urbanization, food systems, education, social inequity, climate change, biodiversity loss, and microbial ecology.^21^ Current development patterns are increasing exposure of ecosystems and populations to climate hazards^22^ and zoonoses, which comprise the bulk of newly emerging infectious diseases.^23^, ^24^ A holistic approach that recognizes dependencies, along with their complex and cascading interactions and scales, will allow for improved prediction, preparation, and adaptation to climate change.^25^ Such an approach can also contribute to food security, reduce costs, better sustain ecosystem services, improve risk communication, strengthen health systems, and ultimately reduce the number of lives lost.^25^, ^26^

### 2.2 Key climate hazards

Rising temperatures, changing patterns of precipitation, and rising sea levels are just some of the climate impacts directly and indirectly affecting public health in New York State. Warming overall means high temperatures that were once extreme or rare become more common, with increases in average maximum temperatures; the number and intensity of extreme heat days; warmer nights that prevent the human body from cooling off after a hot day; and the number, length, and intensity of multiday heat waves. These extremes all exacerbate pre‐existing conditions and disproportionally affect vulnerable populations.^17^ In addition to changes in extreme temperatures, average temperatures are also rising, which can contribute to further health challenges through cumulative impacts that have not been well quantified. Emerging research recognizes that more moderate temperature increases also damage health, while rising average temperatures are expected to increase mortality.^27^ Rising temperatures also contribute to air and water quality degradation.

Changing patterns of precipitation include more intense storm events and higher variability for both dry and wet conditions. Increases in rainfall intensity, or cloudburst events, can overwhelm drainage systems, exposing more New Yorkers to floodwaters. Rising sea levels increase tidal and storm surge flooding, which can also expose more New Yorkers to floodwaters. These types of flooding can cause drowning, injuries, and displacement, along with subsequent physical and mental health effects.^28^, ^29^, ^30^, ^31^

Chapter 2, New York State's Changing Climate, presents data on observed trends and projected changes in these and other climate variables. Section 3 describes their impacts on human health and safety in detail.

### 2.3 Nonclimate factors

Many nonclimate factors can affect public health and safety. Examples include population vulnerability, adaptive capacity, community resilience, and extent and pace of adaptation. In addition to direct climate hazards, this chapter examines health care systems, policies, and costs; availability and equity of health data; the role of pre‐existing conditions; pandemics such as COVID‐19; and effective use of community engagement and communications. It is difficult to determine the exact scope and magnitude of health impacts for each nonclimate factor, because health impacts unfold across different spatial and timescales and within a complex system of social, economic, environmental, and individual factors.^17^

While climate change is a global problem, its public health impacts largely occur at a regional or local level where the capacity to react or adapt varies.^32^ The U.S. public health infrastructure consists of a set of federal, state, and local agencies, with lead responsibility for each city or county often residing at the local level.^33^ New York State's decentralized public health system creates a challenge for local public health departments to prepare for climate hazards. Local health department jurisdictions vary in how they perceive the impacts of climate change and their capacity to address these impacts, highlighting the need for sustained support for programs on local climate change preparedness.^32^

New York's limited health policies and the lack of, and inequality in access to, health data are additional factors that can negatively affect health. These challenges continue as limited state and municipal finances (e.g., due to issues connected with the COVID‐19 pandemic and its cascading consequences^20^—refer to Section 5.4) constrain climate‐related health responses. Even with improvements to climate and health policies and health data management, challenges remain in data justice and understanding and reducing the disparate impacts of climate change on various communities. Issues pertaining to New York's public health infrastructure are discussed in Section 5.2.

The costs of climate impacts on health care include direct care costs such as emergency department visits and hospitalizations as well as other expenses associated with medical care such as prescriptions, home health care needs, and lost wages. Evidence of climate‐sensitive health costs incurred in U.S. populations suggests that these financial stressors are already affecting individuals, families, employers, and both public and private health insurers. Section 3.4.7 explores this topic further. Economic impacts of displacement, loss of affordable housing, interruptions to food and clean water access, and other social burdens are discussed in the Society and Economy chapter.

Several pre‐existing conditions, including obesity, diabetes, high blood pressure, and pregnancy, place individuals at higher risk of heat‐related morbidity and mortality. Other populations are also disproportionately affected by extreme heat. These groups include those with increased exposures to heat and those who work outdoors or in unconditioned spaces, those who are unhoused, those with lower adaptability to heat, and those with energy insecurity at home, regardless of their work or other health conditions. Section 4.5 examines these considerations thoroughly.

Climate hazards (singular events such as Superstorm Sandy as well as compounding ones such as heat waves and Canadian wildfires) during any pandemic, including COVID‐19, can amplify health risks for all. Moreover, climate hazards combined with COVID‐19 are environmental justice issues. For example, in communities with elevated social vulnerability, COVID‐19 infection rates were higher.^34^ These same communities also experience the urban heat island effect. COVID‐19 illustrated that a pandemic coinciding with extreme heat results in multiple health risks, with low‐income communities and communities of color experiencing a disproportionate share of the health burden due to the ongoing impacts of structural racism.^35^ The pandemic also revealed limitations within public and private health systems, which suddenly had to cope with escalating climate challenges, pre‐existing comorbidities such as asthma, and the COVID‐19 pandemic simultaneously. This topic is discussed further in Section 5.4.

Lack of community involvement and adequate support resources can also inhibit effective adaptation and planning, resulting in negative health outcomes. Communities require knowledge and understanding of the local impacts and risks associated with climate change, including clear knowledge about how a health condition or outcome can be linked and/or exacerbated by climate health impacts.^36^, ^37^ This is termed “whole community resilience” or a “whole of community” approach and is discussed in Section 5.3.

New York State's health care and public health infrastructure has a large role to play in promoting and implementing climate adaptation and resilience. Yet, stakeholders need knowledge of local climate hazards, vulnerability, adaptive capacity, and coordination to protect public health. In New York, the involvement of public health professionals in climate change planning is evolving. Policies, programs, and frameworks to address this issue are discussed in Section 5.2.

Because climate change and climate‐sensitive disasters can substantially affect health, it is becoming increasingly essential to link disaster risk reduction to climate change adaptation to address the complex and increasing risks to public health.^38^, ^39^ Prioritizing specific actions to address climate hazards and climate‐related health risks is also important. These actions can include^39^:

- Addressing environmental and social determinants of health relevant to climate‐sensitive hazards.
- Increasing preparedness and response to both climate change and climate‐sensitive disaster risks.
- Developing joint climate information services to inform health programs and services.
- Integrating information services, risk communication, and early warning systems.
- Strengthening health systems’ resilience to facilitate joint adaptation and risk reduction actions for health protection.

### 2.4 Equity and climate justice

In New York State, the climate justice aspects of climate change—including how historical inequities contribute to greater health harms and how and where climate justice initiatives might alleviate those harms and intercept ongoing injustices—are beginning to be recognized and brought into focus by a wider audience, along with concerns surrounding health equity (the opportunity to realize one's full health potential regardless of social circumstances).^4^ However, disease and health burdens and benefits are not equitably distributed. This inequitable distribution depends on many factors, including economic, social, and environmental factors that vary across groups and locations.^40^

Climate‐sensitive health threats pose particular risk to people who lack reliable access to affordable health care. People with chronic health conditions, disabilities, and dependence on support programs are particularly vulnerable and experience greater stress in accessing care.^41^, ^42^, ^43^ Access to care is an ongoing challenge for these community members; climate events simply amplify their challenges when the health service system is disrupted, transportation infrastructure is interrupted, power outages occur, hospital evacuations or facility damage occur, or caregivers are displaced.^44^ Such disruptions can have long‐term effects on how and whether vulnerable populations can access and use local health services.^45^

### 2.5 Indigenous communities

In New York State, the population of those who identify in the census category “American Indian and Alaskan Natives” is nearly 150,000, with more than half of that population living in New York City.^46^ The National Indian Health Board (NIHB) identifies climate change harms to general well‐being as including those impacts that compromise or eliminate traditional ways of life, cultural sites, and sources of subsistence.^47^ The climate‐related health challenges identified by NIHB parallel those identified by the U.S. Global Change Research Program.^48^ Specifically, NIHB categorizes climate change impacts on Tribal health in four ways^47^:

- Those affecting health directly, such as extreme heat, air pollution (including wildfire smoke), and extreme weather.
- Those that spread disease, such as vector‐borne, waterborne, and food contamination.
- Those that destroy or disrupt food supplies, causing or exacerbating hunger, malnutrition, and other conditions.
- Those that disrupt well‐being, causing mental health problems.

NIHB also describes health disparities within Tribes, including higher rates of diseases (respiratory and diabetes) and underfunded health care systems. It estimates resources to be less than half of the actual need. NIHB also notes high poverty and joblessness in more rural and isolated locations, increased vulnerability to harm, and reduced options for care and good health practices. For example, on a national level, some Tribes live in areas with few grocery options, making traditional foods (foraged and hunted) even more important. All these health challenges could be aggravated by climate change. Although beyond the scope of this assessment, recognizing Tribal differences calls for developing specific approaches for each community given varying climate‐related risks, differing spatial contexts, and Tribal norms.

### 2.6 Opportunities for positive change

Responding to climate change impacts in a thoughtful, inclusive manner can deliver opportunities to improve health and safety outcomes for New Yorkers. For example, greenhouse gas reduction and climate resilience strategies frequently have multiple immediate health benefits.^49^, ^50^ Transitioning away from fossil fuel combustion in homes, vehicles, and electric power generation can lead to indoor and outdoor air quality benefits that translate into immediate and long‐term positive health outcomes, depending on such factors as the proximity of communities to legacy sources and the type(s) of fuel being replaced.

When planned and well‐integrated with community and public sector efforts, addressing climate change also brings opportunities to plan actions to raise awareness of health risks, offer near‐term coping resources, participate in the development of hazard mitigation strategies as they relate to health outcomes, and frame adaptation strategies—including training health professionals, monitoring health indicators, and tracking public awareness of risks in a “whole of community” approach. There are many other opportunities to simultaneously address longstanding injustices in health care delivery, community engagement, and resource allocation to historically underserved populations, including those in formerly redlined areas. Opportunities for positive change are noted throughout this chapter and summarized in more detail in Section 6.1.

## 3 IMPACTS AND ADAPTATION STRATEGIES

Climate hazards are worsening existing health conditions, increasing the incidence of diseases (both communicable and noncommunicable), and exposing people to extreme weather events such as heat waves, storms, floods, drought, and wildfires.^51^, ^52^ These hazards include climate impacts in other locations that affect New York State.^53^ Climate change impacts on health and safety are commonly classified as either direct or indirect impacts. Direct impacts can include death, injury, disease, mental health impacts, and other health issues (such as toxic exposures directly linked to a disaster). Indirect impacts are mediated through ecosystems and human systems and can include air pollution (including wildfire smoke); increases in food‐, water‐, and vector‐borne illness; and unmet primary health care needs following a climate event.^51^

This section describes direct and indirect health and safety impacts associated with three main categories of climate hazards: temperature (e.g., means, extremes, seasonal variations), precipitation (e.g., heavy rainfall, hurricanes, other extreme storms and resulting floods, droughts), and sea level rise. A fourth subsection discusses additional impacts that result from a combination of these three hazards and, in some cases, additional climate factors. Each impact discussion also introduces strategies to adapt and build resilience.

### 3.1 Temperature

Projections indicate that the frequency, intensity, and duration of extreme heat events will increase in the coming decades.^54^ The human body is sensitive to heat exposure, including higher ambient heat and humidity. Extreme heat events can harm anyone, even the young and healthy.^55^ Anyone exposed to extreme heat without even a few hours of relief every day is at higher risk for hospitalization or death from hyperthermia.^56^

Health risks from changing temperature are not uniformly distributed. The urban heat island effect is an important driver of extreme heat‐related health effects in dense urban settings. “Urban heat islands are urbanized areas that experience higher temperatures than outlying areas because the built environment (buildings, roads, and other infrastructure) absorbs and re‐emits the sun's heat more than natural landscapes such as forests and water bodies.”^57^ Temperatures can be 1–7°F higher during the day and 2–5°F higher at night in urban areas than in outlying areas. Even within urban areas, there can be temperature differences associated with income and structurally racist land use policies. For example, research has documented that northern Manhattan and South Bronx are hotter than high‐income areas of New York City.^58^, ^59^ (Refer to the Housing Policy and Health case study for more information on redlining practices.)

While extreme heat events pose a direct threat to all New Yorkers, they are especially dangerous for vulnerable populations. Moreover, heat‐related illnesses are often misclassified as other conditions during diagnostic coding (e.g., coding a patient for respiratory illness instead of heat exhaustion).^60^, ^61^

#### 3.1.1 Impacts of temperature

In the United States, extreme heat is responsible for more deaths than any other extreme weather‐related event.^62^ Under a high emissions scenario, climate model projections from 2041 to 2070 across the eastern United States showed that an 8.1°F increase in summer average apparent temperature could occur, triggering an additional 11,562 deaths annually for people ages 65 or older.^27^ (This result comes from the A2 emissions scenario, which comes from an older set of scenarios and involves warming close to, but not quite as high as, the SSP5‐8.5 very high emissions scenario used in this assessment and described in New York State's Changing Climate.) Also, a 5.95°F increase in minimum apparent temperatures (one way of adjusting the actual temperature to account for humidity) could trigger 8767 excess deaths each year in the eastern United States.^27^

Recent modeling studies in New York City showed that the median number of annual projected heat‐related deaths will range from 167 (with high adaptation) to 3331 (with no adaptation) in the 2080s, compared with 638 heat‐related deaths annually between 2000 and 2006.^63^ New York City's 2023 Heat‐Related Mortality Report notes that heat‐exacerbated deaths that occurred on days with temperatures at or above 82°F “show an upward trend in the past decade.”^64^ While some populations (such as athletes and members of the military) actively work toward heat acclimation, others do not, so there are differential risks associated with the level of acclimation.^65^, ^66^

Direct impacts from rising temperatures include cardiovascular and respiratory illnesses, allergies, kidney damage, water‐ and vector‐borne diseases, water contamination, infectious diseases, foodborne diseases, medication efficacy and sensitivity impacts (certain types of medications may exacerbate heat‐related cardiovascular illnesses),^67^, ^68^, ^69^, ^70^, ^71^ and mental health outcomes, as well as psychotic and neurotic outcomes, homicides, and suicides.^72^ A recent study in New York State examined the association between ambient summer temperatures and emergency department visits/hospital admissions. The study found positive relationships between summer high temperatures and acute heat stress, dehydration, cardiovascular illness, and acute kidney failure.^73^, ^74^, ^75^ Associated heat stress can also increase adverse pregnancy outcomes, including preterm delivery, low birth weight, and stillbirth.^76^, ^77^, ^78^, ^79^ Physical work capacity and motor‐cognitive performance can be reduced by heat exposure, with downstream consequences for productivity and increased risk of occupational health problems.^80^

##### 3.1.1.1 Cardiovascular illnesses

Extreme heat exposures are definitively linked to higher rates of cardiovascular disease‐related emergency department visits.^81^ Elevated temperatures stress the body's cardiovascular and respiratory systems and can lead to severe heat‐related illness if the cardiovascular system fails to properly thermoregulate internal body temperature. Excess deaths during heat waves are overwhelmingly cardiovascular in origin (ischemic heart disease, acute myocardial infarction, and congestive heart failure mortality) and occur predominantly in older adults.^82^, ^83^

While all people are vulnerable to heat‐related illness, those living with reduced cardiovascular function and pre‐existing cardiovascular disease are at a greater risk for morbidity and mortality during extreme heat events. Individuals with cardiovascular disease have reduced capacity for blood to flow to the skin both at rest and during exercise, which limits their heat tolerance.^84^

##### 3.1.1.2 Respiratory illnesses related to air pollution

Rising temperatures, combined with changes in humidity, wind speed, and mixing height, can have a negative impact on air quality.^85^ Recent research suggests that increasing temperatures and high air pollution may act synergistically such that the risk of health effects is greater than the sum of their individual effects.^86^ In other words, climate warming may exacerbate air pollution‐related morbidity and mortality burdens even if anthropogenic emissions of air pollutants decline.

One of the most temperature‐sensitive and health‐relevant air pollutants is ground‐level ozone, which is pervasive in New York State and throughout the Northeast. A secondary pollutant, ground‐level ozone is formed when nitrogen oxide and volatile organic compounds react in the presence of heat and sunlight. Higher ambient temperatures cause more ozone to form and also increase natural (biogenic) emissions of certain volatile organic compounds produced by plants; these compounds are ozone precursors.^87^ Ground‐level ozone can irritate the respiratory system, aggravate asthma, and damage the lungs. In addition to acute respiratory effects, exposure to ground‐level ozone and other air pollutants (such as particulate matter) causes premature deaths, hospital visits, and lost school days. One study noted that costs from respiratory disease hospitalizations attributed to extreme heat varied by region.^69^

As the climate changes, many thousands of additional ozone‐related deaths could occur across the United States each year. One study used 2030 emissions to project that increases in average daily maximum temperature of 1.8–7.2°F (1–4°C) and mean daily 8‐h maximum ozone of 1–5 parts per billion would result in “tens to thousands of additional ozone‐related premature deaths and illnesses per year and an economic burden of hundreds of millions to tens of billions of U.S. dollars” (in 2010 dollars).^88^ Higher global temperatures and greater solar radiation are expected to increase ground‐level ozone concentrations in the Northeast.^88^ As a result, more areas within the state could exceed National Ambient Air Quality Standards (NAAQS) established for ozone, negatively affecting human health.

As wildfire risk heightens across North America with rising temperatures and periodic drought, exposure to wildfire smoke—including particulate matter and other volatile organic chemicals—will increase.^89^, ^90^, ^91^ Although wildfires are not a predominant climate‐related hazard within New York State, and they are not projected to become substantially more prevalent in the state during this century, smoke from increasingly large fires elsewhere can travel long distances and reach New York.^54^ For example, Canadian wildfires in 2023 worsened air quality to very unhealthy or hazardous levels in multiple regions of New York State.^92^, ^93^ In June, a statewide air quality advisory was in effect for fine particulate matter (PM_2.5_, or particles less than 2.5 micrometers in diameter) due to the Canadian wildfires.^53^, ^94^ More details on the respiratory effects of wildfire smoke in New York are described in Section 3.2.2.2.

##### 3.1.1.3 Kidney damage

Uncompensated heat stress occurs when hot conditions exceed the human body's cooling ability and a person cannot maintain a steady temperature. Prior research theorized that 95°F (35°C) was the limit of uncompensated heat stress in humans, but research in real‐world conditions suggests this threshold is more likely between 86°F (30°C) and 87.8°F (31°C).^95^, ^96^ These temperatures refer to wet bulb temperature, and are not specific to kidney stress. This lower heat stress threshold, combined with strenuous physical activity, can put certain people at risk for multiple episodes of heat stress, which can trigger pathophysiological responses—including dehydration and rhabdomyolysis—that result in kidney damage or, potentially, kidney failure.^97^, ^98^ Evidence has shown that those who have higher levels of exertion, such as athletes and workers performing physical labor, experience kidney injury due to heat stress.^99^, ^100^, ^101^

##### 3.1.1.4 Waterborne diseases

Increasing temperatures influence the incidence and prevalence of waterborne diseases. Evidence suggests that higher ocean and ambient air temperatures increase the availability and growth of pathogens, such as *Campylobacter, Salmonella*, and *Vibrio*, that cause waterborne disease outbreaks.^102^, ^103^ High temperatures are also associated with increased outbreaks of diarrheal diseases with specific impacts on children.^104^

Waterborne disease outbreaks depend highly on water and sanitation infrastructure. If areas lack these systems, or if climate hazards overrun these systems, outbreaks could occur more frequently.^105^ The association between increasing temperatures and waterborne disease outbreaks from rural water systems or contaminated private wells is not well known.^106^, ^107^, ^108^ However, a combination of rising temperatures and cascading events from other climate hazards, such as heavy precipitation, could increase the rate of waterborne diseases.^109^

##### 3.1.1.5 Foodborne diseases

Increased temperatures can affect food safety. For example, higher temperatures can increase the prevalence of pathogens (including *Salmonella, Vibrio*, and fungi that produce mycotoxins) in produce and meat or fish products. Additionally, increased temperature facilitates the spread of pathogens on contaminated foods, which may increase incidences of gastrointestinal illnesses (e.g., norovirus, hepatitis A).^110^, ^111^ Evidence shows that increases in temperature are directly related to gastrointestinal infection hospitalizations in New York State.^112^ A literature review on the effect of climate change on *Salmonella* in New York identified a handful of studies specifically linking the foodborne pathogen to increasing temperatures.^113^

Water temperatures are increasing along with ambient air temperatures throughout New York's coastal and inland waters.^54^, ^114^, ^115^ Higher surface water temperatures create more suitable environments for harmful algal blooms (HABs) and other microbes.^116^ HABs have been observed with increasing frequency in the Northeast. These blooms can contaminate shellfish, kill fish, and cause illness in people and pets who eat contaminated seafood or become exposed during recreational swimming.^117^, ^118^ As HABs become more frequent, the risk for marine biotoxins in shellfish becomes higher.^119^ Coastal communities have elevated exposure to pathogens in food harvested from warming coastal waters. For example, Indigenous communities and local baymen cultures still rely on subsistence harvesting in waterways. Their lifestyles and livelihoods are threatened by water quality issues due to warming waters.

##### 3.1.1.6 Medication efficacy and sensitivity impacts

Warmer temperatures can affect medication efficacy and complicate chronic disease treatment. For example, psychotropic drugs (including antidepressants, antianxiety medications, beta blockers, stimulants, antipsychotics, and mood stabilizers) can interfere with body temperature regulation; these are a particular concern for older adults.^120^ People who take antipsychotic medications are at particular risk of heat stroke and neuroleptic malignant syndrome^121^ during periods of extreme heat.^122^ Poorly ventilated rooms can increase this risk. Moreover, those with mental health issues may lack awareness of their own risks because of medication use. Certain medications also cause drowsiness or inability to operate a vehicle, making travel to a cooler environment risky or impossible.^123^ People who do not perspire normally, including diabetics and those taking medication for Parkinson's disease, are also more vulnerable to heat. Relatedly, insulin is heat sensitive and must be stored at specific temperatures.

##### 3.1.1.7 Physical activity

Physical activity reduces the risk of all‐cause mortality, cardiovascular disease, type 2 diabetes, and several cancers.^124^ People are less likely to engage in physical activity when it is either very hot or very cold.^125^, ^126^, ^127^ A study of bike share usage in New York City found that as temperatures increased, daily hours and distance ridden substantially increased but then declined above 78.8–82.4°F (26–28°C).^126^

##### 3.1.1.8 Sleep loss

Sleep is essential for both physical and mental health. Several lines of evidence link higher temperatures with sleep disruptions, which can worsen mental health outcomes.^128^ Climate change is projected to unequally erode sleep, further widening inequalities.^129^ Those in warmer climates lose more sleep per degree of temperature rise.^129^ Older adults, pregnant people, and low‐income residents are more affected than others.

#### 3.1.2 Adaptation strategies for temperature‐related impacts

The following sections show how New Yorkers can access resources to cope with rising temperatures. These resources include new or improved tools, frameworks, and data for community planning; public cooling centers; training and planning for emergencies; cool roofs and urban greening; building codes for maximum temperatures; personal heat monitoring strategies; and improved governance, health risk communication, and early warning systems to improve public awareness and municipal responses in advance of extreme heat events.

##### 3.1.2.1 Tools, frameworks, and data for community planning

Tools and frameworks can help public health professionals and communities prepare for climate change impacts and improve resilience. For example, in 2018, researchers designed a statewide Heat Vulnerability Index to identify heat‐vulnerable regions and populations in New York State.^130^ The index can inform long‐range planning and help communities direct adaptation resources where they are needed most. The index assigns a score to each census tract in the state based on four characteristics: language vulnerability, socioeconomic vulnerability, environmental and urban vulnerability, and vulnerability and isolation of older adults.^130^

State and local data sources are also useful for identifying and framing climate health risks. For example, New York City provides an annual report on heat‐related illness and mortality,^75^ and the New York State Department of Health (NYSDOH) provides county‐level heat and health profile reports describing temperature trends, heat‐related health effects, areas with populations most vulnerable to heat, and available adaptation resources.^131^ These reports, which are accessible online, identify heat indices, language and other socioeconomic vulnerabilities, and areas of isolation among older adults—a group particularly vulnerable to heat.^131^ Other data tools for tracking heat and health‐related data include the Environmental and Health Data Portal,^15^ the Heat Stress Tracker,^15^ the National Environmental Public Health Tracking Network program,^132^ the Extreme Heat and Health in New York State StoryMap,^133^ and Heat.gov.^7^

##### 3.1.2.2 Cooling centers

Cooling centers can be effective temporary adaptations to extreme heat; however, they are underused and unequally distributed within New York City and across the state.^134^, ^135^, ^136^ Access to cooling centers is also limited for those dependent on public transportation.^136^ In addition to improving household access to affordable air conditioning, which is the best option (refer to Section 4.9), New York could benefit from establishing more, and better, public cooling centers and other publicly accessible adaptations to extreme heat events. A 2015 survey found that 12% of sampled adults reported traveling to a public cooling center if they could not keep cool at home.^137^ The environmental justice organization WE ACT's 2020 research offers helpful suggestions on how to improve cooling center programs across New York City.^138^ Their suggestions include establishing comprehensive criteria that sites must meet to become cooling centers, spreading information about site locations and features, training staff to identify signs of heat stress in site visitors, extending hours of operation, supporting public transit access, and creating a welcoming atmosphere with programming.^139^

##### 3.1.2.3 Emergency preparedness

Effective preparation for extreme heat emergencies includes training at the municipal level and securing energy and backup resources for heat hazard mitigation. New York City has adopted these strategies and others as part of its emergency preparedness planning.^140^, ^141^

##### 3.1.2.4 Outdoor physical environment

Modifications to the outdoor physical environment, such as improved and increased tree canopies, greenspaces, and green roofs, can help moderate the urban heat island effect and reduce heat gain^142^ while providing cobenefits, such as natural habitats for urban pollinators. Rochester's Climate Change Resilience Plan incorporates urban forestry to reduce the urban heat island effect in the city.^143^ Buffalo's Rain Check program, while focused on installing green infrastructure for stormwater management, acknowledges the cobenefits of that infrastructure for also reducing the urban heat island effect.^144^ New York City's Green Infrastructure program (administered by the New York City Department of Environmental Protection and the New York City Department of Parks and Recreation) and Green Roof Tax Abatement are examples of efforts to add cooling vegetation to the urban environment. However, these urban greening strategies could contribute to “green gentrification,” raising housing prices and forcing longtime residents out of their home neighborhoods.^145^

##### 3.1.2.5 Building codes for maximum indoor temperatures

New York State does not currently have code requirements for maximum indoor temperatures, although many jurisdictions do regulate minimum allowed indoor temperature to prevent illness and protect infrastructure from freezing (e.g., New York City Administrative Code 27–2029, 2017; Syracuse Code of Ordinances 27–54, 2023). However, high indoor temperatures are a particular concern, especially in dense urban housing that lacks adequate cooling. Studies have found that apartments do not cool down at night in accordance with outdoor temperature; instead, indoor temperatures stay dangerously high after a hot day and remain consistently warmer than the outdoors.^146^ For additional information on built environment characteristics as related to temperature, refer to the Buildings chapter.

##### 3.1.2.6 Personal heat monitoring

Personal heat monitoring enables individuals to make more informed decisions about heat exposures.^147^ The Occupational Safety and Health Administration and the National Institute for Occupational Safety and Health offer practical ways to monitor exposure, including guidelines and a phone app.^148^ More broadly, the Convergence program, initiated in North Carolina and expanded in 2022 to cover the majority of the East Coast,^149^ offers weekly forecasts of wet‐bulb (“real feel”) globe temperature, which can improve personal decision‐making on heat exposure. In 2019, the New York City Department of Corrections issued recommendations on daily temperature monitoring in its correctional facilities, along with and short‐ and long‐term actions to reduce heat risks.^150^, ^151^

##### 3.1.2.7 Communications and community

The public health impacts of climate change will take place mostly at the local level, which means that local knowledge of climate hazards, vulnerability, adaptive capacity, and coordination will be necessary to protect public health. Climate policy and planning alone are not sufficient for the community‐level action needed to adapt to the changing climate.^152^ Targeted communications and tight‐knit communities can provide adaptive benefits that increase resilience to extreme heat impacts from climate change.

Despite health science demonstrating the substantial morbidity and mortality risks of elevated temperatures, not everyone may be fully aware of or have received communication on the health threat posed by extreme heat. Without knowledge of personal risk, populations are less likely to take precautions. Adaptation solutions can improve community awareness of extreme heat risks. When communicating about extreme heat health threats, it is critical to acknowledge that distinct subpopulations have differing risk perceptions. A national study in 2019 found that low‐income neighborhoods and those with larger populations of color have higher risk perceptions than high‐income neighborhoods with more white residents, a pattern “consistent with vulnerability differences across these populations.”^153^ A survey conducted in New York City found that while low‐income individuals were less likely than those with high incomes to be aware of heat warnings, they were more likely to be concerned that heat could make them ill and that climate change would affect their health.^137^ Understanding an audience's risk perception can help policymakers tailor messaging accordingly.

It is also important to define daytime and nighttime temperature thresholds for activating emergency heat measures. Recognizing that heat‐related illness in New York State occurs at lower temperatures than existing warning thresholds, regional offices in New York and Vermont reduced the heat advisory criteria from 100°F to 95°F based on NYSDOH recommendations; even lower criteria are being considered or are in place in some areas for particularly vulnerable populations.^73^ A 2019 study found that lowering the activation threshold for New York City's heat emergency plan was associated with reduced heat‐related illnesses during hot days, compared with a counterfactual scenario in which the threshold did not change.^154^

Improving social connections can reduce heat‐related health risks for some isolated groups.^155^ Public health awareness campaigns, online resources, training and communications from health service providers, and broad public messaging before and during extreme heat events can improve awareness and motivate health‐protective behavior change.

Involving community members in heat‐related data collection or science projects can also be an effective way to improve local awareness of extreme heat and heat islands while setting the stage for subsequent interventions. For example, campaigns conducted in the Bronx and Yonkers in 2021 and in Manhattan and Brooklyn in 2022 encouraged community members to collect temperature data throughout the urban environment.^156^

Innovations in communications include work by King County, Washington, which established a multicultural comics series over the course of several years to better communicate health risks and coping strategies related to extreme heat, subsequently expanding the series to include a range of health issues.^157^ Prior to developing this resource, King County sought ways to help community members understand their heat‐related health risks and their options for addressing those risks at home.^158^ The U.S. Environmental Protection Agency (EPA) recognized King County as a winner of its “Let's Talk About Heat Challenge” for its innovative approach to raising awareness and protecting public health.^159^ King County's website offers easily accessible resources in multiple languages with associated culturally relevant examples. An example of a King County comic demonstrating tips to stay cool in the heat is shown in Figure 7‐3.

**FIGURE 7‐3:**
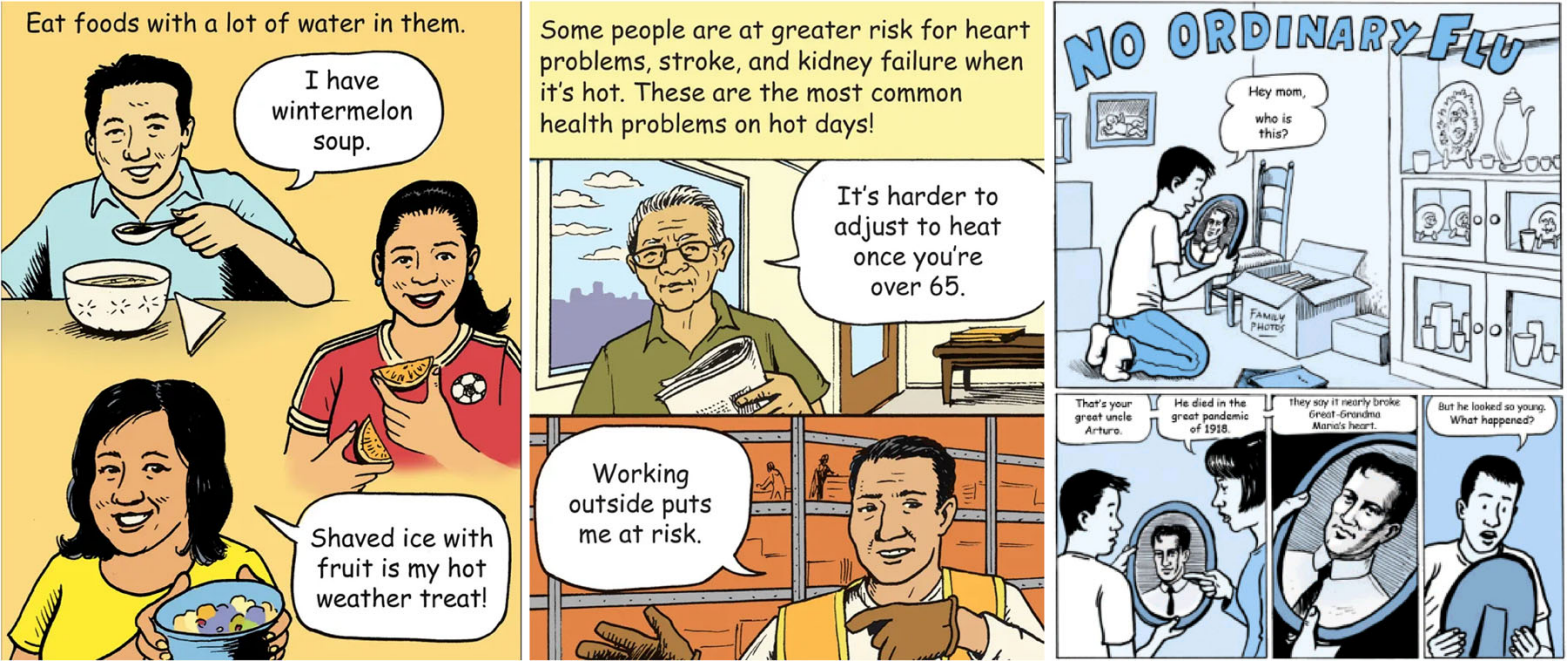
Examples of comics used by King County, Washington, to improve the awareness of health risks and coping strategies. Figure from Li‐Vollmer.^160^

##### 3.1.2.8 Work conditions

High ambient temperatures will affect how outdoor and heat‐exposed occupations function in the future. Adaptation efforts to modify working hours and address heat risks in both outdoor (e.g., military,^66^ agricultural,^161^ postal^162^) and indoor (e.g., schools) working environments^158^ can improve population health in the face of rising temperatures.^158^ More specifically, Morris et al., in their 2020 review of solutions to mitigate occupational heat stress, found that environmental cooling, cooling garments, physiological adaptation, and personal cooling interventions (e.g., cold water immersion) were successful in preventing heat stress.^163^ Traditional occupational practices to reduce heat stress, such as hydration, seeking shade, and reducing work during peak temperatures, can also help workers^164^ and athletes; however, customizing actions for workplaces and workers will be most effective in preventing occupational heat stress.

Overheated workers can lower body temperature by stopping work, removing equipment and extra clothing, immersing themselves in cool water, raising legs and reclining, and replacing electrolytes and rehydrating.^165^ Workers can self‐monitor personal heat stress by understanding how to lower body temperature and knowing which methods (e.g., fans) are effective and when.^166^ However, some workers could be reluctant to mention heat stress to their employers due to concerns about losing their jobs, being paid less, or not being taken seriously. The Occupational Safety and Health Administration has worked to establish worker guidelines, but many undocumented workers and other workers with less negotiating power remain at risk.^167^, ^168^

##### 3.1.2.9 Regulatory and policy solutions

Climate change could make it necessary to develop new policies for heat safety. It could also trigger existing heat safety policies. The New York State Public High School Athletic Association has heat safety procedures for high school sport contests and practices: a heat index less than 79°F permits normal participation in activities, while every five‐degree increase up to 96°F requires actions to minimize thermal stress. Above 96°F, all outside activities cease, and only inside activity with air conditioning is permitted.^169^ Athletes are particularly vulnerable to heat stress; due to their perceived health and often young age, they may be more likely to play outdoor sports when temperatures are beyond safety thresholds.^170^, ^171^, ^172^ In New York City, the Department of Education has developed guidelines for playing outdoors in cold weather (i.e., activities can be prohibited if it is snowing, there is ice on the playground, or the wind‐chill factor is below 0°F) and hot weather (i.e., school staff should limit outdoor playtime between the sun's peak hours in temperatures above 95°F, reduce intensity of activities, provide easy access to water and encourage drinking often, pay special attention to those susceptible to heat‐related illness).^173^ Due to regional variations, some researchers recommend lower heat warning thresholds based on wet bulb globe temperature.^174^ NYSDOH regulates some types of buildings (e.g., congregate living facilities), and can help increase awareness of heat impacts on people with pre‐existing conditions and set expectations for the monitoring and management of those conditions and the buildings in which those individuals reside.^175^ (Refer to the Buildings chapter for a more detailed discussion of adaptations related to indoor temperature.)

To counter extreme heat, municipalities could consider programmatic interventions (e.g., schedule changes) and environmental interventions (e.g., tree planting) to improve thermal comfort at settings intended for physical activity.^125^ Additional strategies could be necessary to safeguard public transit riders who experience higher temperatures while waiting for subways and buses.^176^ High temperatures have been shown to reduce transit ridership; however, bus stop shelters and nearby tree shade lessen these ridership losses on public bus routes.^177^, ^178^

### 3.2 Precipitation

Extreme precipitation events relevant to New York State include heavy rain, snowfall, cloudbursts (sudden downpours that drop large amounts of rain in a short time), hurricanes and other tropical storms, and extratropical cyclones such as nor'easters. These events can cause direct injuries and deaths, flooding, and subsequent exposures to pathogens and hazardous substances. Intense precipitation has historically caused flooding in many areas across New York.^54^ Three types of flooding have direct health impacts: (1) pluvial, which refers to flooding of built environments such as streets; (2) fluvial, which refers to flooding from overtopping of rivers or streams; and (3) coastal, which refers to storm surge and tidal flooding. Pluvial and fluvial flooding stem from extreme precipitation events. Coastal flooding stems from sea level rise, which is often compounded by coastal storms (refer to Section 3.3). A fourth type of flooding from rising groundwater is not addressed here.

As New York State's Changing Climate explains, higher air and water temperatures result in more evaporation and precipitation, which can lead to more intense storms and severe floods. The Northeast has experienced some of its heaviest downpours (extreme 2‐day precipitation events) and flooding over the past few decades. Cloudburst rainfall intensities continue to surpass historic rainfall intensities and extend flooding well beyond designated flood zones, particularly in cities.^179^ In addition to heavy rainfall, the intensity of North Atlantic hurricanes has increased since the mid‐1990s, although trends in frequency and longer‐term trends in intensity are harder to discern.^180^, ^181^

Projections show that changes in precipitation patterns in the Northeast will increase winter‐spring rainfall and cause a larger share of annual precipitation to occur in heavy events.^54^, ^182^, ^183^ Projections developed for this assessment using a blend of intermediate and very high greenhouse gas emissions scenarios (SSP2‐4.5 and SSP5‐8.5) indicate that all regions of New York State can expect to experience an increase in the number of days with more than 1, 2, or 4 inches of precipitation through the 2080s.^54^ However, even though New York is considered a water‐rich state and is likely to get wetter over time, water shortages could occur more frequently and with greater intensity with the changing climate. Projections indicate that late summer, short‐duration droughts will become increasingly frequent and more intense during this century,^54^ affecting water supply for agriculture, ecosystems, and energy production. Heavy precipitation events are likely to occur more frequently but result in less replenishment of water sources and soil moisture, especially if the dry periods between events lengthen and temperatures increase.

#### 3.2.1 Heavy rainfall and flooding impacts

New Yorkers’ health can be directly affected by heavy precipitation events, storms, and contact with floodwaters. Heavy precipitation events and associated flooding can cause death, injury, and illness; worsen underlying medical conditions; and adversely affect mental health.^184^ Other flood‐related health impacts include trauma, electrocution, pathogen exposures, mold exposures, carbon monoxide poisoning, and hypothermia.^185^, ^186^, ^187^ Toxic floodwaters can cause dermatitis, rashes, burns, headaches, fatigue, abdominal pain, fever, decreased appetite, nausea, sore throat, and eye irritation.^188^ In addition, mental health challenges have been found to be associated with increasing temperatures, extreme weather events, and loss of livelihoods and culture.^22^ (Refer to Section 3.4.1.)

Flooding can limit access to health services, which affects those who need chronic condition care and emergency response support.^189^, ^190^ (Refer to the Hospital Floodwall Retrofit case study to learn more about impacts and adaptation strategies at one New York State hospital.) Flooding can also affect electrical systems and cause power outages for days or weeks, which can limit transport to medical facilities, pharmacies, and other destinations, as well as the ability to conduct essential errands—particularly in buildings with elevators.^191^ Power outages affect people with impaired mobility, those whose medications (such as insulin) need refrigeration, and those who rely on air conditioning. This combination of impacts is particularly challenging for older adults who have diabetes. (Refer to the Energy chapter for further discussion about the impacts of climate change on energy systems.) In New York City alone, more than 60,000 people age 65 and over live in public housing, and more than one‐third of them have diabetes.^192^

Vulnerability to flooding is greater for those who work in emergency response and reconstruction, as many fatalities are associated with recovery activities. The risk is also greater for people who have disabilities, those with compromised immune systems and illnesses that require frequent medical treatments (e.g., dialysis), pregnant people, and newborns. People with limited financial means to flee or recover afterward are at greater risk for illnesses, injuries, or mortality from flooding.^193^, ^194^

Researchers in New York State have conducted research on unequal social vulnerability to Superstorm Sandy flood exposure,^195^ and the New York State Energy Research and Development Authority conducted an assessment in 2017 of population vulnerability to climate change.^195^, ^196^ Findings reveal that indicators of vulnerability to flooding include race, income, age, education, language, pre‐existing health conditions, and housing status (unhoused or living alone).

##### 3.2.1.1 Drowning

Drowning is the most common cause of death from flooding nationwide.^197^ As recently as 2021, 13 people in New York City^198^ and five more people in the rest of New York State^199^ drowned due to flooding associated with Hurricane Ida.^198^ Basement apartments can flood during cloudburst events, displacing people already living in vulnerable situations. While such units offer relatively affordable housing for low‐income renters, an estimated 100,000 basement apartments in New York City are illegal, and many lack sufficient means of exit.^200^

##### 3.2.1.2 Electrocution and other injuries

Flooding is linked to electrocution and other injuries. An investigation of 52 injury deaths in New York City related to Superstorm Sandy identified incidents of unintentional injury, including electrocution, transportation‐related harms, cuts and piercing, poisoning, suffocation, falls, burns from fires, and hypothermia.^201^ Additional deaths were caused by carbon monoxide exposure and asphyxiation linked to generator use. Some of these events were categorized as “secondary deaths” that occurred because of the loss of infrastructure and utility services.^202^

##### 3.2.1.3 Mold exposures and respiratory illnesses

The increasing frequency of storms and heavy precipitation events, along with coastal flooding, affects the dampness of buildings and could increase the severity of mold exposure.^203^ Mold exposure can trigger lower respiratory symptoms, including wheezing, coughing, and shortness of breath.^204^ Cleanup and reconstruction activities after Superstorm Sandy increased the risk for lower respiratory symptoms linked to mold or damp environment exposures and other respiratory irritants.^204^

Climate change also modifies the availability and distribution of mold and fungal‐derived allergens. After thunderstorms, higher atmospheric concentrations of fungal spores can increase the future availability and distribution of fungal‐derived allergens.^203^, ^205^

##### 3.2.1.4 Hazardous substances and toxic chemical exposures

Extreme precipitation can lead to flooding that carries toxic materials into the environment and nearby communities, introducing new health risks and amplifying existing problems.^206^ For example, flooded hazardous waste sites and disturbed toxic reservoirs can disperse pollutants into both the natural and built environment. Industrial facilities, agriculture, and utilities have historically been situated near bodies of water, which makes it more likely that floods could remobilize, transport, and deposit hazardous substances.^207^ These areas will likely be at risk of continued contamination from climate‐induced extreme precipitation events.^208^

Floods carrying toxic materials frequently affect vulnerable communities, many of which are located near contaminated sites. New York City has clustered public infrastructure, water pollution control plants, waste transfer stations, energy facilities, and heavy manufacturing in six Significant Maritime and Industrial Areas.^209^, ^210^ These areas are mostly located in low‐income communities and communities of color. Storm surges and floods could disperse pollutants in these coastal communities.^209^, ^210^

Heavy precipitation events such as Hurricane Harvey in Texas in 2017 and Superstorm Sandy in New York in 2012 exposed communities to a wide range of hazardous substances, including polycyclic aromatic hydrocarbons and organochlorine pesticides.^211^, ^212^ After Sandy, researchers examined how toxic chemicals from EPA Superfund sites could have migrated via floodwaters from sites such as Newtown Creek and the Gowanus Canal into residential areas in New York City.^213^

Flooding after Sandy also led to an increase in residential oil spills in the state. The Bureau of Toxic Substance Assessment investigated approximately 2600 incidences of residential oil spills after Sandy (the annual average is 350 investigations) (Muscatiello, N, 2022, New York State Department of Health, Personal communication). Residents were largely responsible for managing their own spills due to the scale of the disaster.^214^, ^215^, ^216^, ^217^, ^218^

Storm‐related pollution from Sandy also included “widespread pollution of the Hudson River and New York Harbor by a variety of toxic chemicals, including petroleum and fluids from cars and boats; contaminants from flooded subways, roads, parking lots, and tunnels; and contaminants washed from shoreline industrial sites, as well as commercial and residential buildings.”^219^ Poorer harbor water quality is a detriment to surrounding communities, aquatic life, and natural coastal environments.^220^

##### 3.2.1.5 Pathogen exposures and waterborne illnesses

Extreme precipitation events can generate floodwaters that contain pathogens hazardous to human health, including raw sewage, bacteria, and viruses. Ingesting floodwaters can trigger waterborne illnesses, including upset stomach, intestinal problems, headache, and flu‐like discomfort. Contaminated drinking, bathing, or recreational waters can also increase exposure to infectious waterborne diseases.

The older combined sewer systems common in the Northeast can be vulnerable to heavy precipitation. As the Water Resources chapter explains, more than 10 million New Yorkers—more than half the state's population—live in communities served by combined sewer systems.^221^ These systems use one pipe to collect sewage, industrial wastewater, and stormwater runoff, and transport these combined wastewaters to a municipal treatment facility. The treated wastewaters are then discharged into a local water body. When system capacity is exceeded during a heavy precipitation event, a combined sewer overflow (CSO) occurs as untreated sewage is released to local waterways. These discharges can diminish the quality of recreational waters and sources of drinking water. CSO discharges pose risks for human health, including gastrointestinal illness and skin infections from direct exposure to contaminated water and asthma exacerbations due to aerosolized lung irritants and other pathogens.^52^, ^104^, ^222^ In an Ohio study, direct contact with or proximity to aerosolized CSO effluent increased the risk for childhood emergency department visits for gastrointestinal illness.^223^

Animal and human waste from affected wastewater treatment plants can also infiltrate water systems, increasing the incidence and prevalence of pathogens associated with gastrointestinal illness and other infections.^224^ Evidence from various states, including New York, shows that heavy precipitation events led to increases in gastrointestinal illnesses hospitalizations, with more cases among children.^112^, ^222^, ^225^ This relationship is particularly strong for communities served by municipal water systems that do not disinfect (typically smaller systems).^226^

Model simulations in a Buffalo study showed that a future (2070–2099) 50‐year storm will generate CSO volumes comparable to a current (1970–1999) 100‐year storm due to the more intense precipitation and resulting runoff. Overall, projections show Buffalo's CSO volumes increasing by 11%–73% in 2070–2099 compared with 1970–1999 for different climate‐related storm events, potentially increasing the likelihood of waterborne disease transmission.^227^

##### 3.2.1.6 Foodborne illnesses and food safety and security

Flooding can inundate crops, impair food storage, and disrupt distribution centers and food transport in New York and more broadly. These impacts can affect livelihoods and food security across New York State, which in turn can have consequences for health.^228^ Further, food storage and delivery are at risk due to increased power outages and weak infrastructure.^229^ Some areas in the state are at particularly high risk from flooding disruption. For example, 95% of the food for Manhattan, which supports more than 1 million residents, is delivered by truck via a few bridges and tunnels.^230^ (Refer to the Agriculture and Transportation chapters for further discussion about climate change impacts on New York's food production systems and transportation infrastructure, respectively.)

Another way that flooding affects food safety is through runoff (e.g., from industrial and agricultural sources and CSOs) that carries pollutants into local water bodies. These contaminants can harm or kill fish directly and bioaccumulate in certain species. People who consume contaminated seafood over long periods of time face elevated risks of chronic health effects that range from endocrine disruption to cancer.^231^, ^232^

#### 3.2.2 Drought impacts

The factors that influence drought severity are complex and can last for months or years, making droughts especially insidious as a climate hazard. A range of factors determine whether a drought will occur. These factors include local geology, impermeable surface extent, water usage patterns, and adaptability of water supply systems. Climate change is not expected to increase the risk of extended, multiyear droughts in New York State, but shorter‐term seasonal droughts lasting from weeks to months could increase.^54^ From a public health preparedness and response perspective, drought should be considered a chronic or “low rise” natural event rather than an acute emergency (except in the case of saltwater intrusion risk such as that in Louisiana in 2023).^233^

In New York State, drought conditions are monitored across 11 regions and four stages of drought^234^:

1. **Drought watch**. The public is encouraged to conserve water, but no statewide mandatory use restrictions are in place.
2. **Drought warning**. Voluntary water conservation measures are intensified, and public water suppliers implement drought contingency plans.
3. **Drought emergency**. Mandatory water restrictions may be imposed. The public may need to use alternative water sources.
4. **Drought disaster**. Water use is further restricted. Emergency legislation and disaster declarations may be made.

The more severe stages have public health implications, as they coincide with greater fire risk, higher incidence of mosquitoes carrying West Nile virus, and agricultural losses (products and jobs).^235^

Droughts present several health implications for New Yorkers and can affect human health in many ways, both directly through decreased water availability and indirectly through impacts on low‐income areas.^236^ Health risks associated with drought include increases in overall mortality; gastrointestinal illness; respiratory disease; stress; and mosquito‐borne disease (West Nile virus); as well as nutritional deficiencies; food insecurity; and concomitant health effects due to higher prices, decreased income, or shifting expenses to purchase water.^237^ Additional health impacts associated with droughts include foodborne and waterborne diseases; airborne and dust‐related diseases; illnesses related to exposures to toxins; mental health effects; and other health effects related to wildfire, including the impacts of smoke.^238^ These vulnerabilities are amplified for more marginalized populations, such as Tribal communities, incarcerated persons, or displaced and migratory communities.^239^, ^240^, ^241^, ^242^

##### 3.2.2.1 Waterborne illnesses from water shortages

Water shortages from rain shortfalls or drought conditions increase the need for crop irrigation, as well as the need for energy production for cooling in higher temperatures. These needs can compete with other water needs, leading to shortages of drinking water and reduced drinking water quality.^236^ Water conservation measures implemented during a drought could increase public health risks, particularly in congregate care facilities, nursing homes, hospitals, prisons, and schools.

Several areas of New York State have unique water quality and availability challenges due to local geology. For example, Long Island depends on groundwater from an aquifer that is at risk for increased saltwater intrusion during prolonged droughts.^243^ Heavy rainfall during drought conditions can concentrate pathogens and contaminants in water sources. The contaminant burden on treatment plants also increases, as do the risks associated with HABs, which increase in frequency and severity during droughts. HABs can contaminate water systems and make local drinking water unsafe.^244^, ^245^ (For further insights on HABs, refer to the Water Resources and Ecosystems chapters.)

During extremely dry or wet conditions, impacts on water quality typically affect private, individual (residential) water supply wells first. In New York State, more than 1 million homes and several million residents and visitors are served by private wells.^246^ While public water supplies are regularly tested for a variety of contaminants, inspected, and maintained, private wells are the responsibility of the owners. If private well owners are not diligent about upkeep, contamination risks could increase, putting those served by these wells at risk.

##### 3.2.2.2 Respiratory illnesses related to drought and wildfires

Hot and dry conditions can contribute to air quality impairments through the mobilization of wind‐blown dust particles as well as potential changes in emissions patterns of ozone precursors (biogenic emissions) produced by certain types of vegetation.^247^ Wildfires, fueled by increasingly prevalent drought conditions, also considerably reduce air quality. Wildfire smoke contains a variety of chemical and particulate components.^91^ Many air pollutants in wildfire smoke aggravate cardiovascular and pulmonary disease, and strong associations have been found between exposure to wildfire smoke and exacerbation of asthma, chronic obstructive pulmonary disease, bronchitis, and pneumonia.^248^, ^249^

While wildfires are not expected to become markedly larger or more frequent in New York State during the 21st century,^54^ more intense fire seasons elsewhere in the United States and Canada can diminish air quality in New York through long‐range transport.^250^ Throughout June 2023, for example, the New York State Department of Environmental Conservation (NYSDEC) and NYSDOH issued statewide air quality alerts due to Canadian wildfires (refer to Figure 7‐4).^53^, ^94^ During this period, PM_2.5_ measurements in New York City reached the historically high daily average of 460 micrograms per cubic meter, a concentration that EPA's Air Quality Index deems hazardous to human health.^251^ Asthma‐associated emergency department visits increased across the state, with greatest impacts in Eastern Lake Ontario and Central regions and among older children and young adults (ages 10–29).^252^ Moreover, air quality alerts in July 2023 continued to warn residents of public health risks associated with wildfire smoke exposure.^253^ Recommended preventative measures included closing schools, canceling outdoor events, and warning residents to stay inside.^92^, ^254^

**FIGURE 7‐4:**
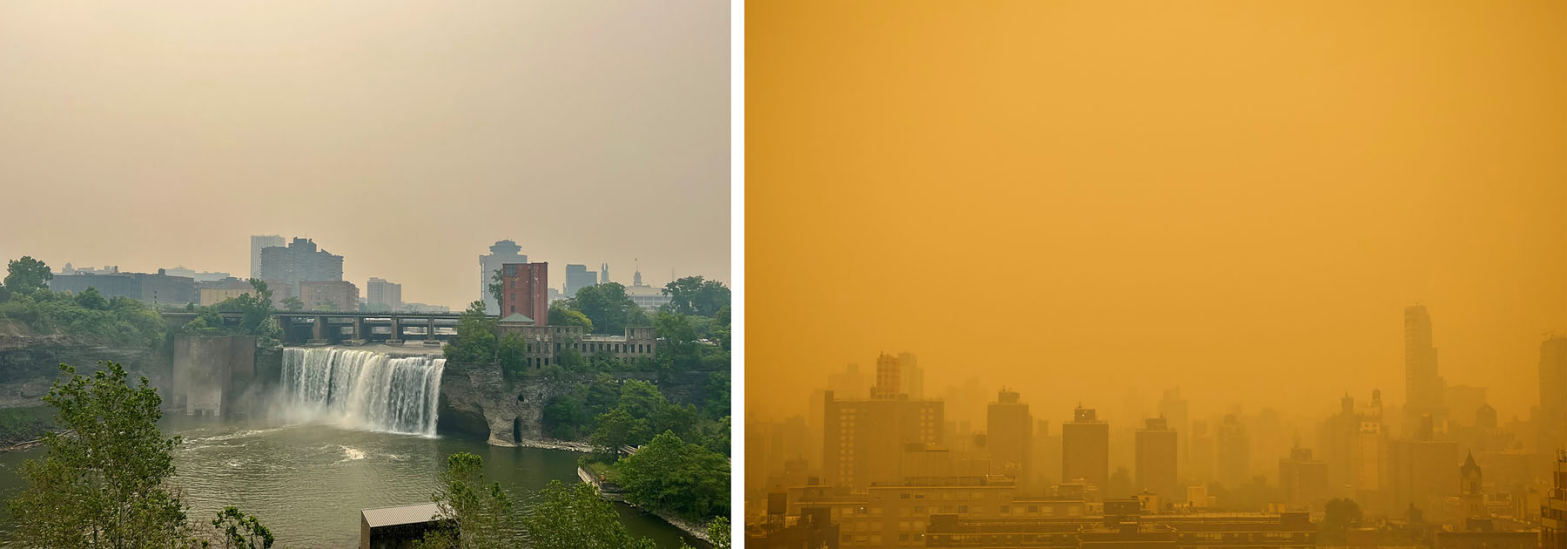
Smoke from the June 2023 Canadian wildfires in Rochester (left) and New York City (right). Photos by Dena Krichinsky, ERG (left); iStock (right).

A global study of short‐term health impacts of wildfire emissions found significant connections between all‐ and specific‐cause mortality and wildfire smoke.^255^ All‐cause respiratory outcomes were also significantly associated with wildfire smoke, while cardiovascular‐related effects appeared to be most significant several days after exposure. A 2023 study on long‐term health impacts of nonoccupational wildfire exposure found that while evidence was limited, associations were observed with “mortality, morbidity, shorter height of children, reduced lung function, and poorer general health status.”^256^ A second 2023 study focused on estimating economic losses via wildfire‐induced health impacts from 2012 to 2014.^257^ In the New York metropolitan areas, this estimate included 86 annual premature deaths and $0.78 billion in economic loss.^257^

#### 3.2.3 Adaptation strategies for precipitation‐related impacts

Adaptation strategies for precipitation and flooding are most effective when implemented before, during, and after extreme events. These strategies include community‐scale projects to manage water detention, as well as efforts at individual homes to divert water volumes and reduce exposure to flooding. Additional strategies include implementing flood warning systems, securing access to shelter, coordinating community responses, modifying infrastructure, instituting and improving flood risk management practices, taking individual action, improving the built environment, and pursuing managed retreat. Issues pertaining to managed retreat are discussed in Section 3.3.2.3.

##### 3.2.3.1 Flood warning systems

Timely and effective communication about flood hazards is critical to help communities understand available resources and act swiftly. It is also important to assess areas recently affected by extreme events to better understand public health exposures. For example, during Hurricane Ida, traditional gray infrastructure practices were quickly overwhelmed by the volume of rain.^258^ Future precipitation is expected to be more intense, which makes it even more critical for New Yorkers to understand their individual exposures and the flood risk in their neighborhoods. Following the lessons learned from Hurricane Ida, New York City Emergency Management implemented the Notify NYC warning system which, among other notifications, alerts basement apartment dwellers to seek higher ground when extreme rainfall is expected.^259^ While the program requires users to opt in to receive those notifications, it represents the type of immediate warning system that can encourage people to get out of harm's way.

Recent studies have shown that early flood warning systems have major benefits that greatly exceed their costs by allowing people to act before disaster occurs.^260^ One study found that the basic architecture of a flood early warning system requires four components: disaster risk knowledge, forecasting, dissemination and communication of information, and preparedness and response.^260^ It is important for the early warning system to be “people‐centered,” with active community participation from design to implementation. Another study found that flood information tools codeveloped with engineers and local stakeholders achieved important outcomes, including building a greater shared understanding of flood risks, facilitating the exchange of ideas, providing a framework for development, encouraging interaction among scientists and end users, and providing accessible and understandable information to end users.^261^

##### 3.2.3.2 Access to shelter

Access to evacuation shelters prior to flooding is an important adaptation strategy. However, this strategy relies on the National Weather Service providing ample warning in advance of coastal storms and nor'easters, so that people have enough time to travel to shelter locations before extreme weather events take place. Shelters are less helpful for unexpected cloudbursts that occur quickly and provide little time to prepare, but can be useful for those receiving evacuation notices for basement apartments. However, cloudburst events are flooding areas previously less affected by extreme precipitation, where shelter access may be limited. Recognizing the need for a more comprehensive understanding of flood vulnerability, New York City is in the process of developing a Flood Vulnerability Index for release in 2024.^262^

Both forecasted flooding and unexpected cloudburst events introduce health challenges, even for individuals who relocate. Those who relocate may forfeit or compromise continuity of care, risk exposure to illnesses in the community, and suffer financial impacts. (Refer to the Buildings and Water Resources chapters for related preventative measures.) Access to shelters also involves equity concerns, and those who rely on public transportation or who live far from a shelter may not have access to these resources.

##### 3.2.3.3 Community coordinated response

Health service providers, emergency responders, shelter operators, and community‐based organizations remain the primary resources for community members during flood events. In urban and rural areas, immediate resources remain limited. Recent analysis of New York City 311 (nonemergency services line) call data related to stormwater flooding shows a 1‐ to 4‐day response time across the city's five boroughs.^263^, ^264^

Community‐level hazard mitigation efforts still rely on municipal leadership, given that understanding of these measures is still developing. Examples such as the New York City Department of Environmental Protection Cloudburst program^265^ and New York City Housing Authority's Connected Communities program,^266^ a joint project between the New York City Housing Authority and New York City Department of City Planning, introduce smaller‐scale, community‐specific interventions, but these measures are in development and lack longitudinal data on efficacy.

Emergency management and social services offer individual and family support for recovery from flood events. Community‐based organizations provide material support and help community members navigate city programs for recovery. Private companies offer mold remediation and other cleanup services, although associated costs and market capacity limit access to such resources. Additionally, some frontline and environmental justice communities are creating their own tailored adaptation plans. For example, the Akwesasne (Saint Regis Mohawk) adaptation plan speaks to multiple impacts of heavy rainfall and recommends education, community, and collaborative responses.^267^ In 2015, the environmental justice organization WE ACT, based in New York City, led tabletop community exercises around complex climate disasters that resulted in the Northern Manhattan Climate Action plan.^268^ Any substantive adaptation planning can benefit from connecting to housing and land use policy reform to reduce risks.^269^

##### 3.2.3.4 Infrastructure modifications

Actions for wet and dry floodproofing of homes and businesses, as well as more extensive home raising or relocation of mechanical systems, can help property owners reduce their exposure to future flooding.^270^ In addition, improvements to water treatment and wastewater collection and treatment systems could reduce illness associated with flooding and heavy precipitation events.

##### 3.2.3.5 Flood risk management

Hazard mitigation activities, such as those that address living with floodwater or insuring against flooding, help community members reduce flood‐related health risks at home. Resources include the National Flood Insurance Program. This program offers a resource for homeowners, renters, and small businesses to reduce the financial impacts of flooded properties, although the costs of premiums are considerable and vary greatly by structure, which excludes some vulnerable populations. The latest version of the Federal Emergency Management Agency's Risk Rating (2.0)^271^ improves risk communication, but does not address concerns about flood insurance affordability. Individual assistance requests through the Federal Emergency Management Agency or the U.S. Department of Housing and Urban Development can help some homeowners or renters with flood insurance claims; however, the average grant size and the wait time to receive funds do not provide sufficient immediate support.

##### 3.2.3.6 Individual action

In most extreme precipitation events, community members will need to alter behaviors to avoid flood areas, which can have indirect health impacts. Behavior changes could include avoiding subways, buses, and certain roads; relocating households from basement and ground floor units (recognizing the challenge given the availability and affordability of housing alternatives); or relocating from properties altogether.

##### 3.2.3.7 Built environment

Some built environment adaptation and resilience solutions jointly address urban heat and flood risks while improving public health. For example, green infrastructure, urban park space, and porous pavement lessen flooding in urban environments and reduce the retention of incoming solar radiation and accumulation of heat. Such interventions also improve mental health by facilitating increased access to nature.^272^ Quantifying the health‐related benefits of these land cover interventions is an important step in demonstrating the value of these adaptation actions for addressing climate hazards, especially in urban settings. Recent work on the New York City Open Streets Program introduces opportunities for these shared benefits.^273^, ^274^

### 3.3 Sea level rise

A 2021 report by NYSDEC calls sea level rise “the most directly observable effect of climate change in New York.”^275^ Along the coast of New York and in the tidally influenced portions of the Hudson River, sea level has risen by more than 1 foot since 1900 and is predicted to increase into the future, with slight regional variations due to local conditions.^275^ Projections developed for this assessment show that sea level will rise along the New York coastline and in the tidal Hudson by 7–12 inches by the 2030s, 12–21 inches by the 2050s, and 21–41 inches by the 2080s, compared with a 1995–2014 baseline.^54^ Sea level rise makes storm surge and flood risk acutely worse during storm events. Over longer periods, it can render land uninhabitable due to regular high‐tide flooding and permanent inundation. According to the National Oceanic and Atmospheric Administration's latest national sea level rise report^276^ and projected sea level rise in New York State, homes and businesses will experience more tidal and storm surge flooding, which will put more New Yorkers at risk.

#### 3.3.1 Impacts of sea level rise

The 2015 New York City Panel on Climate Change report^277^ identified at least seven pathways for storms and flooding to impact New Yorkers’ health, including several that are similar to those related to flooding (refer to Section 3.2). These include:

- Direct exposure to storm hazards (such as drowning).
- Exposure to secondary hazards related to utility outages and sheltering in place in inadequate housing after the storm.
- Exposure to secondary hazards, including contaminated drinking water, contaminated floodwaters, and mold and moisture in housing.
- Evacuation, which increases risks—particularly for those relying on others and/or relying on life‐saving devices.
- Population displacement and disruption of services.
- Mental health effects from traumatic or stressful experiences during and after the storm.
- Health and safety risks from cleanup and recovery activities.

People settled in low‐lying coastal areas could face increased soil and water salinification, flooding risk, and local emergence of infectious diseases. For example, drinking water wells and septic systems are potentially at risk from rising saline water tables.^278^

If health care and other lifeline infrastructure (e.g., utilities; fuel sources; food distribution; communications; water supply; sewage treatment; and transportation, including subways and airports) are located in areas exposed to sea level rise and storm surge, the risk of service interruption and long‐term failure increases.^279^ Land where toxic materials are stored and land polluted by these materials (e.g., active and capped brownfield sites) can introduce contaminants into the environment, risking the exposure of nearby populations. CSOs also pose backflow risks when pipes are no longer only covered during high tides.^280^ The Water Resources chapter provides additional details about these impacts.

Displacement via disasters or planned retreat is hugely disruptive.^281^ Impacts are uneven, as people with fewer resources could be less able to relocate. Additionally, certain communities (e.g., some Tribal communities) have strong familial ties to the land that could make them less inclined to relocate.^282^

#### 3.3.2 Adaptation strategies for sea level rise‐related impacts

Adaptation strategies to address sea level rise include raising public awareness, coordinating community responses, pursuing managed retreat, and decommissioning assets.

##### 3.3.2.1 Public awareness

Awareness of expected sea level rise impacts across New York State varies, which is consistent with research on perceptions elsewhere.^283^, ^284^ Beyond the flooding issues addressed earlier in this chapter, sea level rise introduces broader challenges for community awareness and resulting actions. A central challenge is that sea level rise is a “next generation” problem, as modeling uses long time horizons such as 2050, 2080, 2100, and 2150.^285^ This perception can make it difficult for community members to consider near‐term decision‐making, even as rising waters already affect daily life. Communities such as Edgemere and Broad Channel in New York City already experience high‐tide flooding due to sea level rise. Montauk is conducting community outreach to address retreat in its Coastal Assessment and Resiliency Plan.^286^ Following Superstorm Sandy, Oakwood Beach in Staten Island participated in a major community buyout program.^287^ However, there is a lack of clarity on how rising waters could affect migration patterns toward or away from coastal areas.^288^, ^289^ Effective engagement is essential for minimizing the public health impacts of climate change, including sea level rise.^290^, ^291^

##### 3.3.2.2 Community coordinated response

Communities that consider the interrelationships between critical infrastructure functionality and human health impacts can better adapt to climate change.^292^ Coastal community planning departments in New York State could prioritize evaluating the exposure of critical infrastructure to sea level rise in concert with health departments, as this infrastructure affects public health services and population exposures. For example, Hunts Point, New York City's food distribution center, lacks coastal barriers sufficient to address long‐term sea level rise or even near‐term storm surge.^293^

Sea level rise warrants more integrated approaches to understand climate and health impacts. Like many coastal states, New York needs statewide interagency coordinated efforts to cope with near‐term impacts, manage hazard mitigation, and adapt over the long term.^294^, ^295^ However, some local efforts are underway. For example, the New York City Climate and Health Program focuses on integrating health considerations into planning and resilience initiatives to address public health impacts of sea level rise. New York City's recently updated Comprehensive Waterfront Plan also mentions public health. Although this plan does not mention health in the context of sea level rise and related chronic conditions facing many communities, it does recognize that efforts should be made to limit future residential densities in areas with coastal flood risks to prevent scales that cannot be managed.^296^

##### 3.3.2.3 Managed retreat

Long‐term managed retreat from certain flood‐prone areas is an important adaptation for chronic flooding from heavy precipitation or sea level rise. The expected need for managed retreat will be a challenge for many coastal areas of New York State. Researchers anticipate a greater need for managed retreat programs across the United States, even as the population increases in coastal cities.^297^ This process requires public health engagement, given its impact on social cohesion, mental health, and economic stability (as evidenced by the Staten Island buyout program following Superstorm Sandy).^287^ Analysis shows increasing inequities in managed retreat as political motivations and cost‐benefit logic lead to decisions that disproportionately displace low‐income or overburdened communities while investing more resources in sea level rise preparedness and recovery for higher‐income areas.^298^, ^299^, ^300^ Conversely, communities receiving populations that are retreating from flood‐prone areas must also prepare for such in‐migration, which raises risks of gentrification and further displacement.^269^

A lack of coordinated efforts to integrate public health into the planning for managed retreat and/or other strategies to address sea level rise could isolate vulnerable populations. Communities can benefit from understanding the near‐term impacts of sea level rise on their locations, even as they plan for the long term. Moreover, health professionals can improve outcomes by developing coping strategies and planning for mid‐ and long‐term transitions in coastal areas and inland flood‐prone areas. Limited shared knowledge makes it difficult to enact broader coping strategies.^301^, ^302^, ^303^ Recent efforts in the Edgemere neighborhood of Queens, led by The Nature Conservancy, offer important lessons for community cocreation of managed retreat processes.^304^

##### 3.3.2.4 Decommissioning

While many organizations are planning for climate adaptation, few have addressed the effects of long‐term higher sea level on their operations, including the possible need to decommission assets.^279^, ^305^, ^306^ These assets could include energy, transportation, or industrial infrastructure (e.g., septic systems, capped brownfields, roads, fuel tanks) that need to be removed or relocated. Decommissioning intentionally removes modifications to a natural environment and requires risk assessment beyond the visible site areas. Decommissioning areas of permanent inundation will be an important strategy in caring for the health of New Yorkers. This process requires collective responsibility (whole of community) to ensure the total removal of nonadaptive built investments or assets. When areas reach tipping points (e.g., chronic or permanent inundation) and functional, sustained use is no longer reasonable, assets in these areas no longer serve their purposes. Examples include buyout areas where residential occupancy is no longer possible due to flooding. New York's post‐Sandy Staten Island buyouts, as well as buyouts in inland fluvial areas, meet those criteria. However, decommissioning requires more than simply removing residential structures. It requires removing the systems that served it (e.g., propane tanks, septic tanks, electrical and gas lines to the property) and restoring the site to support natural ecological systems. Not doing so means leaving these systems exposed to salt water and risking further water contamination as those former assets corrode or erode.

### 3.4 Intersecting hazards

Additional health and safety impacts can result from a combination of the hazards described above (temperature, precipitation, sea level rise) and climate factors, such as increased carbon dioxide concentrations in the atmosphere. These include impacts on mental health, conflict and violence, airborne allergens and asthma, food production, vector‐borne diseases, other infectious diseases, population displacement, and health care costs. This section summarizes current knowledge about these impacts and their projected occurrence in New York State in the decades ahead.

#### 3.4.1 Mental health

Climate change can affect mental health in multiple ways. Impacts include medication and clinical management considerations,^307^ disaster‐response aspects,^308^ and climate grief.^309^ These impacts occur after heat or extreme weather events, as well as indirectly after climate‐related economic loss, threats to health and well‐being, migration, and conflict.^17^, ^22^, ^310^, ^311^, ^312^

Palinkas and Wong categorize mental health consequences following three types of climate‐related events: (1) extreme weather events lasting for days, such as hurricanes, floods, wildfires, and short‐duration heat waves; (2) subacute weather events lasting for months or years, such as droughts and long‐duration heat waves; and (3) environmental changes lasting to the end of this century and beyond, such as higher temperatures, sea level rise, and a permanently altered and potentially uninhabitable physical environment.^311^ Psychological stress and anxiety around the existential threat related to climate change could be one of the greatest long‐term mental health outcomes associated with the changing climate.^311^, ^313^

Climate change has been shown to affect mental health across the age spectrum. Among a demographically representative sample of American adults aged 18 and over, more than half of the sample reported being moderately or very afraid of climate change.^314^ Older adults and young people have been found to be particularly vulnerable to the mental health impacts of climate change.^315^, ^316^ Among children, climate change has been linked with high levels of concern and stress^315^, ^317^; after climate events, they typically demonstrate more severe distress^318^ and higher rates of PTSD than adults.^319^

Heat can exacerbate pre‐existing mental health issues. Elevated temperatures have been linked to violence and different types of mental health problems, with the strongest evidence for elevated suicide risk. There is emerging evidence that people living with mental health challenges may be at increased risk from heat‐related morbidity and mortality.^320^

Flooding events can cause acute stress and other important mental health problems stemming from both short‐term impacts (e.g., injuries, disease) and long‐term effects (e.g., population displacement, homelessness). Direct harm caused by flooding events can be compounded by long‐term disruption of lifeline infrastructure and public services (including impaired delivery of electricity, clean drinking water, waste removal, and public transportation), which in turn can have negative mental and physical health impacts. Mental health impacts from disasters can include PTSD, general distress, anxiety, and other psychiatric disorders. PTSD is the most‐studied effect in the aftermath of disasters^321^ (e.g., after flooding caused by Superstorm Sandy),^322^ but the full extent of disaster‐related mental health impacts is not well studied or understood.

The prolonged experience of drought can trigger important mental health consequences, especially in rural populations.^323^ Drought conditions can impose numerous stressors on people in ways that sometimes overlap. These stressors include loss of livelihood for people in agriculture and other natural resource occupations, diminished social support, and loss of a sense of place and belonging as climate change transforms thriving landscapes into more desolate surroundings (directly or through means such as wildfires).^324^, ^325^ The link between drought and mental health has not been extensively studied to date. The bulk of research has focused on economic mechanisms by which drought affects livelihoods and worsens stress and other mental health outcomes.

Chronic physical and mental consequences and stress from extreme events can inflict and perpetuate long‐lasting trauma. For example, studies have shown that individuals and communities affected by Hurricane Katrina have long‐term mental and physical health concerns decades after the storm, even if they no longer reside in New Orleans or affected areas.^326^

Chronic stress contributes to higher suicide and homicide rates and an increasing need for mental health counseling and training to reduce exposures, even as those resources remain limited.^72^, ^327^, ^328^, ^329^

#### 3.4.2 Conflict and violence

Climate change is a threat multiplier for violence: it increases the risk of collective violence due to already‐existing causative factors.^330^, ^331^ Evidence gathered across geographies and time indicates that extreme weather conditions and warmer temperatures more generally are associated with increased incidence of violence, including interpersonal and domestic violence and possibly child abuse.^330^, ^332^, ^333^, ^334^, ^335^, ^336^ Research indicates a rise in violent crime with rising temperatures, as well as seasonal patterns that show more crime during the summer.^337^ These associations could be caused by increased aggression and discomfort during hot weather, by alterations in routine activities caused by high temperatures, or interactions between other socioeconomic or demographic factors. Recent work by Kondo et al. compared assault cases and found that lack of tree cover, which exacerbates extreme heat, was associated with more gunshot assault in low‐income communities.^338^, ^339^ Additional studies specific to New York State could help identify precise intervention points tailored to local communities.

Other factors associated with collective violence include socioeconomic and political instability, land ownership disputes, poverty, and disparity.^330^ At a global scale, the changing climate is predicted to diminish or alter essential natural resources in many regions, resulting in water, food, and livelihood scarcity and potentially leading to desperation and government challenge, enhancing the risk of conflict.^340^ Climate migration is recognized as one outcome, as the Society and Economy chapter examines in more detail.^341^ While climate‐related conflict cannot be projected precisely because of its complexities, social cohesion and community resilience can have a buffering effect.^330^, ^342^

#### 3.4.3 Airborne allergens and asthma

Climate change is expected to worsen allergy‐related illnesses because allergenic pollen levels are affected by warmer weather, higher humidity, and heightened levels of carbon dioxide in the atmosphere.^343^, ^344^, ^345^, ^346^, ^347^ Climate change can influence production, allergenicity, distribution, and seasonal timing of airborne allergens (aeroallergens)—namely pollen—in several ways. First, warmer spring temperatures cause certain plants to start producing pollen earlier, while warmer fall temperatures extend the growing season for plants such as ragweed that produce pollen in summer and fall.^17^ A longitudinal transect study in the central United States and Canada found that the ragweed pollen season grew longer at nearly every pollen‐counting station examined from 1995 to 2015, with larger increases at more northerly latitudes.^344^, ^348^ Stations in Wisconsin, at the same latitude as New York State, registered 15‐day increases in ragweed season length.^348^ Second, warmer temperatures and increased carbon dioxide concentrations in the lower atmosphere enable certain plants to produce more‐allergenic pollen in larger quantities.^17^ Rising carbon dioxide concentrations, along with warmer temperatures and fluctuating precipitation patterns, also influence plant and fungal species’ development more generally.

Changes in the timing, duration, and intensity of allergy seasons will affect indoor and outdoor air quality and exacerbate asthma and other allergic illness.^17^ This effect coincides with changes in ozone (refer to Section 3.1.1.2) in contributing to the burden of respiratory diseases, including asthma, in New York State. In a New York City (Bronx) study, asthma‐related emergency department visits were highest in December and January and lowest in July, with a spring peak in April and May.^349^ The spring asthma peak was closely associated with increased tree pollen counts, and the asthma increase was likely due to allergic reactions to pollen. Research on New York City hospital admissions for asthma indicates that pollution effects can vary considerably across pollutant type, geography, season, and age.^248^ Moreover, biological or abiotic (i.e., chemical/physical) interactions will affect exposure to and health consequences of allergens.^350^

#### 3.4.4 Food production and nutrition

Climate hazards, such as higher temperatures and extreme weather, reduce food availability (via power outages and other mechanisms) and nutritional quality. Increasing evidence indicates that rising carbon dioxide concentrations negatively affect the nutritional quality of major crops such as rice and wheat, lowering protein, micronutrient, and B vitamin levels.^351^, ^352^

Climate and other environmental changes could reduce the yield of vegetables and legumes, which could have implications for food security. Increasing temperatures, changing precipitation patterns, and greater frequency of extreme events also influence yields of other crops and livestock systems, reduce food quality, and disrupt food storage and transportation networks,^353^ further threatening nutritional status. For example, any flood‐affected food is considered adulterated by the U.S. Food and Drug Administration, and therefore, not fit for consumption. With reduced yields, lower nutritional quality, and inflationary pressures, vulnerable populations already experiencing food insecurity will likely experience more. The growing emphasis on urban agriculture as a supplement to local food sources could serve as an adaptive strategy.^228^, ^354^, ^355^ The New York State Department of Agriculture and Markets’ Farm to School program offers an integrated food program to help stabilize student diets in rural communities while strengthening the farm economy and raising awareness of regional food.^356^ The Agriculture chapter provides a comprehensive review of these and other impacts and adaptations within New York's food production sector.

#### 3.4.5 Vector‐borne diseases

Vector‐borne diseases are mainly transmitted through arthropod vectors (e.g., insects, arachnids), which are particularly sensitive to changes in temperature, precipitation, and weather patterns.^357^, ^358^ Increasing temperatures affect vector development and capacity (survival and reproduction rates), biting rates, and replication of pathogens transmitted by vectors.^359^ Temperature changes can also influence ranges and populations of wild animal vector hosts, including deer, mice, and birds, which can drive the expansion and distribution of vectors such as mosquitos and ticks.^360^, ^361^ Increased precipitation directly affects the abundance and availability of many vectors, particularly mosquitos and ticks. Precipitation affects mosquitoes’ reproductive habits and lifecycles and increases their numbers, which could raise the risk of exposure to vector‐borne diseases among populations where heavy precipitation events and flooding have occurred.^362^

Lyme disease and West Nile virus are New York State's most common tick‐ and mosquito‐borne diseases, respectively. Though West Nile virus was not identified in the United States until 1999 in New York City, it is now the most common mosquito‐borne disease nationwide.^363^, ^364^ Several modeling studies have predicted a geographic increase in West Nile virus cases across New York State and Connecticut.^365^ Specifically, changing temperature, precipitation, and extreme weather patterns are altering the geographic boundaries of suitable vector habitats, increasing the risk of West Nile virus as well as other vector‐borne diseases.^70^, ^365^ While precipitation also has an influence on tick survival and host‐seeking behaviors, other factors—such as temperature and contact frequency with alternative hosts and humans—play a greater role.^70^ A 2019 study recognizes that changes in geographies or seasonality of mosquito‐borne disease (increases, decreases, no changes) depend on varying thermal ranges, and that a more nuanced modeling of thermal physiology coupled with vector, pathogen, and host ecology is needed.^366^

New York has not seen local transmission of certain mosquito‐borne diseases, such as malaria, dengue, and yellow fever, in decades. However, increasing temperatures and decreasing freeze cycles, globalization, and vector‐borne disease outbreaks could encourage the further spread of these diseases.^359^

While Lyme disease accounts for more than 82% of tick‐borne disease cases in the United States,^367^ Anaplasmosis, a nonspecific influenza‐like illness transmitted by ticks, has increased in incidence in New York State.^368^ The Capital Region alone has experienced an eight‐fold increase in cases between 2010 and 2018.^368^ With broadening tick habitats and increasing human populations living near wildlife, a further increase in cases is almost certain.^368^

Common adaptive measures include human behavior changes related to outdoor activities, clothing, and the use of insect and tick repellents. Vector control strategies can also play a role. For example, several of the Tribal Nations in New York have climate adaptation plans that note the need to address stagnant pools resulting from heavy precipitation and poor drainage that serve as breeding grounds for mosquitoes.^267^, ^369^

#### 3.4.6 Other infectious diseases

The past decade has shown an emergence and re‐emergence of many infectious diseases, including COVID‐19, Ebola, and Middle Eastern respiratory syndrome (MERS). While the geographic distribution of infectious diseases has changed due to factors such as travel and globalization, the effect of climate change on ecosystems and economies could raise the risk of some infectious diseases, thus increasing the need for proactive surveillance.^370^, ^371^

Changes in ecological conditions due to climate change are leading to disturbances that affect infectious pathogen characteristics, transmission, hosts, and habitat.^372^ In addition to geographic distribution, overcrowding and poor sanitation during extreme weather events and evolving bacterial resistance are changing the risk of infectious disease associated with climate change.^372^ These hazards affect public beaches and camps, which are regulated by NYSDOH and local health departments.

Several other temperature effects can influence the incidence and prevalence of disease caused by pathogens. For example, rising temperatures can increase the number and distribution of pathogens that produce mycotoxins, which can contaminate food crops.^372^ There is also evidence of an association between increasing temperatures and antimicrobial resistance for certain pathogens.^373^

#### 3.4.7 Population displacement

There is growing evidence that changing environmental and climate conditions trigger migration and displacement.^374^ In the 2020 Atlantic hurricane season, 2.8 million new hurricane and tropical storm displacements were recorded across 17 countries and territories.^375^ Although displacements have not yet been tracked in New York State, evidence shows that displacement followed Superstorm Sandy with extensive buyouts in Staten Island,^376^ and also followed Hurricane Ida as flooded basement apartments in New York City forced residents to flee.^377^ Rebuild by Design and Milliman studied New York City's risks of displacement due to coastal flooding, including approximately 1.8 million people who live in low‐income neighborhoods.^378^ They argue that 40% of the city's residents are at risk of displacement due to coastal storms and rising sea levels. The costs of relocation in expensive housing markets make a challenging situation even more difficult, particularly given the disproportionate impact of flooding on affordable housing.^379^, ^380^

Recent research emphasizes the impacts of displacement on health, including an increasing interest in mental health impacts. A 2020 study illustrates the relationships between population displacement and health.^381^ Another recent study explored displacement's impact on health care access and mental health for residents in New York City, noting increases in emergency department visits and hospitalizations due to mental health.^382^ Although this study focused on gentrification, the issue of displacement remains pertinent. Current research also recognizes the increased vulnerabilities of climate migrants due to physical exposures and financial burdens, as well as the need for health systems to further prepare for in‐migrations.^383^ In 2023, the Substance Abuse and Mental Health Services Administration identified climate‐related disruptions leading to “increased violence and crime, decreased community, and increased social instability.”^384^ For further insights on displacements, refer to the Society and Economy chapter.

#### 3.4.8 Health care costs

The health care costs of climate change include direct care costs (e.g., emergency department visits and hospitalizations), as well as other expenses associated with medical care, such as prescriptions, home health care needs, and lost wages. Evidence of climate‐sensitive health costs incurred in U.S. populations suggests that these financial stressors are already affecting individuals, families, employers, and both public and private health insurers. For example, in a study of costs associated with hospitalizations for heat‐related illness in the United States using the 2001–2010 nationwide inpatient sample, patients of color were at a higher risk of being hospitalized, and Black, Hispanic, and Asian/Pacific Islander patients had higher hospitalization costs associated with heat‐related illnesses.^385^ These patients could have a greater disease and financial burden due to climate‐sensitive events such as extreme heat. The paper emphasizes the financial burdens (hospitalization costs) on nonwhite, older, and low‐income populations due to hospitalizations for heat‐related illness.

The combination of higher hospitalization risk and higher costs for certain vulnerable populations could be indicative of the burden from other climate hazards, as there is often alignment between more vulnerable populations and more climate‐related exposures, such as larger urban heat island effects and more impervious surface contributing to more pluvial flooding. For example, time away from work due to hospitalization (loss of income) due to illness or injury resulting from climate hazards could follow a pattern similar to health disparities observed for certain racial/ethnic groups and low‐income populations related to non‐climate hazard‐associated hospitalizations.^385^ A 2019 study identified $3.1 billion (in 2018 dollars) in health‐related costs in New York and New Jersey from Superstorm Sandy due to 273 deaths, 6602 hospital admissions, and 4673 emergency department visits.^386^

Respiratory health problems can result in considerable economic burdens. The estimated respiratory disease burden attributable to extreme heat during a baseline period (1991–2004) in New York State was 100 hospital admissions, $644,069 in direct hospitalization costs, and 616 days of hospitalization per year.^69^ According to the same study, respiratory hospital admissions due to increasing excessive heat in the next few decades could be two to six times higher in 2080–2099 than the baseline.^69^

Health service providers now experience direct climate‐related cost increases for additional staffing for emergency responses to extreme heat and increases in emergency medical service response costs,^387^ as well as lost productivity and missed appointments during storm and flooding events.^388^, ^389^ Similarly, municipalities now devote additional budgets to health risks associated with energy insecurity in times of extreme heat.^390^ New York City's seniors’ air conditioning program, Get Cool, is one example.^391^

## 4 VULNERABILITY, EQUITY, AND ENVIRONMENTAL JUSTICE

Climate change, health, and equity are intertwined. The impacts of climate change (e.g., higher temperatures, more extreme weather, sea level rise) are felt by all, yet certain populations—particularly people of color and people with low incomes—feel the impacts sooner and more severely due to historical discriminatory policies and ongoing disinvestment.^392^, ^393^, ^394^, ^395^ For these and other vulnerable populations, climate change is a threat multiplier, amplifying existing inequalities and increasing marginalization.^392^, ^396^, ^397^, ^398^, ^399^, ^400^ The differential impacts and vulnerabilities felt by these populations emerge from higher exposure to climate‐related events, higher sensitivity to those exposures (e.g., greater heat sensitivities among children and older adults), and lower adaptive capacities due to constrained or unavailable resources. Several of these differences result from structural racism, including disinvestments and marginalization.^401^

Climate vulnerability refers to the predisposition to be adversely affected by climate‐related health effects.^17^ Differential vulnerability to climate change exists within and across regions and is modulated by social, behavioral, and health context; historical and ongoing patterns of inequity and marginalization; resource and infrastructure access; culture; knowledge; governance; immigration status; and other factors, all of which operate at multiple scales.^22^, ^402^ NYSDOH's Vulnerable Populations Summary has identified seven categories of populations vulnerable to climate change (Table 7‐1).^403^ Many factors can amplify the risk of exposure to climate hazards and negative health outcomes among these groups, as discussed in the sections below.

**TABLE 7‐1:** Populations vulnerable to climate change in New York State.

Category	Examples of affected populations
Age	Older adults, young athletes, young children, infants.
Race/ethnicity	Various groups of people of color, Indigenous people on Tribal lands.
Gender	Women, gender marginalized groups.
Income	People with low income,^404^ especially women, infants, and children.
Health status	People with asthma or other respiratory/pulmonary conditions; people with cardiac problems; people who are heat sensitive, immunocompromised, on dialysis, dependent on medical devices, homebound, disabled, pregnant, in need of nutrition assistance, taking certain medications (e.g., beta blockers); people with other chronic conditions; and uninsured individuals. Refer to Section 4.5 on chronic pre‐existing medical conditions for more detail.
Location	People living near hazardous waste sites that are not well contained; people living near flood‐prone areas; inpatients and others in health care facilities; people living in public housing, naturally occurring retirement communities, coastal areas, and areas served by private residential well water; people living alone, on higher floors of apartment buildings, without air conditioning at home, in urban heat island areas, or in poorer quality housing; and those lacking housing.
Occupation	Outdoor workers (e.g., farmers, construction workers); migrant or seasonal workers; warehouse workers; athletes; workers near heat sources, including firefighters; military personnel; and those not using vector protective measures.
Immigration status	Groups of immigrants who lack stable housing, incomes, and access to care, and/or groups who are impeded in seeking help through language or other resource barriers.
Behavior	Those not using vector protective measures.

*Note*: Categories and examples from New York State Department of Health (2015)^404^; other examples from sources noted throughout this chapter.

### 4.1 Structural racism

The Centers for Disease Control and Prevention (CDC) recognize racism as a serious threat to public health for racial and ethnic minorities.^405^ Additionally, New York City, alongside the American Public Health Association, declared structural racism a public health crisis.^406^, ^407^, ^408^ Structural racism refers to “the totality of ways in which societies foster discrimination, via mutually reinforcing systems of discrimination (e.g., in housing, education, employment, earnings, benefits, credit, media, health care, criminal justice, etc.) that in turn reinforce discriminatory beliefs, values, and distribution of resources”^409^ and is a larger concept than institutional racism or race‐based policies.^410^

A recent study that examined the legacy of systemic racism in housing policy on resident exposure to urban heat in 108 U.S. cities—including Yonkers, New York—found links between historically underserved areas and current lack of tree canopy, degree of impervious surface (which increases floodwaters and increases urban heat island effects), poverty, race, and higher land surface temperatures.^411^ Furthermore, higher land surface temperatures are correlated with excess morbidity and mortality.^412^ Gee and Payne‐Sturges’ stress–exposure–disease framework (2004) helps explain how differential experiences of community stress, exposure to pollutants, and access to community resources may, through psychosocial stress, lead to greater health disparity.^413^

Climate justice necessitates that New York State consider how historical inequities contribute to greater health harms and how and where climate justice initiatives might alleviate those harms and intercept ongoing injustices. Research shows that climate change acts as a risk multiplier, increasing adverse health outcomes—including effects on children's health and health care costs, disproportionate impacts on certain communities, and exacerbated effects of other social determinants of health.^414^ Some communities are beginning to address local climate action in the context of structural racism and its impacts on health and safety. An example of such work is the North Manhattan Climate Action Plan, created with input from community workshops led by WE ACT for Environmental Justice.^268^

### 4.2 Indigenous communities

Connections between the physical environment, cultural traditions such as foodways and medicine, and well‐being highlight the multifaceted impacts of climate change on Indigenous Peoples. For example, climate‐related changes affect the Shinnecock people of Long Island in multiple ways. Changes to surface water and oceans affect the Tribe's shellfish cultivation, and sea level rise and saltwater intrusion affect Tribal lands that provide food and medicine. The Shinnecock face risks from land loss and impacts on food security, as well as reduced harvests from land and water due to increased temperatures and storms. With sea level rise, the Shinnecock lands could become uninhabitable. (Refer to the Shinnecock Nation Marine and Land Farming Adaptations case study to learn more about these impacts and potential adaptation strategies.) The Shinnecock Nation's 2019 climate vulnerability assessment and action plan also identified concerns over increased heat‐related stresses, flood‐induced restrictions on emergency access, risks of pest outbreaks and Lyme disease, and reduced fisheries and shellfish health.^415^ Traditional subsistence fishing and foraging could increase exposure to disease vectors, pathogens, and other contaminants. The decreasing availability of sassafras, huckleberry, shellfish, and pitch pine introduces new risks to traditional food, medicines, and cultural resources such as wampum.^415^

The Akwesasne (Saint Regis Mohawk) climate change adaptation plan emphasizes stress and mental health impacts, limited access to health services, and worsening air quality that could amplify existing respiratory and cardiovascular diseases that already occur due to the Akwesasne Territory's extensive industrial contamination. In 2013, the Saint Regis Mohawk Tribe began the process of identifying sensitive cultural resources that could be affected by climate change. The Akwesasne write, “Mother Earth continues to provide us with sustenance as she has always done since the beginning of time as she was originally instructed by the Creator. The Land, and a defined land base are essential for the survival of our culture, our lifeways and our People. We are not the owners of the soil or the Earth, but we are dependent upon the Earth for our survival. It is our responsibility for its protection so that successive generations of our people and the other Nations of Creation will continue to receive sustenance from the Earth in the same way that we have.”^267^ Their climate adaptation plan organizes hazards, exposures, vulnerabilities, and risks according to systems, such as “Medicine Herbs” and “The Four Winds.”

Through the efforts of its Watershed Resources Working Group and Climate Change Task Force, the Seneca Nation has identified reductions in wildlife and aquatic species and associated risks to traditional food preservation. The Seneca describe how increasing water temperatures in the Allegheny River, Cattaraugus Creek, and Lake Erie pose risks for cultural and environmental preservation. A particular example relates to their food supply. In 2022, the Seneca received partial funding from the U.S. Department of Agriculture to address chronic wasting disease in the Nation's deer population.^416^ Recent modeling of climate impacts shows that efforts to reduce chronic wasting disease could be hindered by climate change, which could reduce the deer population and threaten this subsistence food source.^417^, ^418^

Although not a climate program per se, the Tuscarora Environment Program focuses on residential water access, wetland restoration, food preservation and production, and waste management.^419^ It consistently identifies water security and water quality as health risks exacerbated by climate change, particularly for those not on a public water system. The NIHB also acknowledges these issues and recognizes the spiritual and physical need for water.^420^ Many Tribes also identify the need for resources focused specifically on home weatherization programs, a key element in safe sheltering at home.

Tribal food security may be further threatened by changing growing patterns and availability of First Foods and wild edibles such as wild strawberries, maple syrup, and shellfish, which are traditional food sources.^421^ For Tribes, foods are important not only as calories, but also for where they come from and what they provide. For example, Gakwi:yo:h Farms of the Seneca Nation focuses on food security and food sovereignty in an effort to improve eating habits by drawing on Seneca history and ancestral practices toward food. For the Onondaga Nation, similar efforts focus on the preservation of bison and traditional heirloom seeds.^422^, ^423^ Further, many traditional communities view food as medicine and need certain foods for optimal health. Availability is important, and climate change is affecting when and if these foods can be harvested. Harvesting, medicine, and other traditional practices may be disrupted through climate change impacts, even as some Tribal organizations try to improve productivity and availability. If these challenges are not overcome, climate change will create even greater challenges for Tribal food security and well‐being.

Tribal Nations have delimited lands, some of which may be lost to rising seas. With managed retreat or disaster‐related displacement, some Tribal Nations may be forced to leave their ancestral lands, burial grounds, and local sources for traditional diets and medicinal plants.^424^, ^425^ This type of relocation has major psychosocial consequences, in addition to the material stresses inherent in any relocation. In light of histories of forced relocations, loss of native lands, oppression, and loss of resources, relocations are a particularly compounding issue for Tribes.^426^ Further, given the important social support role of the large, close‐knit, family‐like network in many Tribal Nations, relocation that separates community members brings unique threats to Indigenous communities. These challenges occur in the context of a paucity of mental health resources and services for Indigenous communities.^425^, ^427^, ^428^, ^429^

With chronic poverty and limited health resources, Indigenous communities also suffer disproportionately from high blood pressure and diabetes. In the context of COVID‐19, the New York City Department of Health and Mental Hygiene conducted its first study on the health conditions of the population of 100,000+ Native American/Indigenous Peoples within the city.^430^ Key findings highlight “lower prevalence of access to health care and a higher prevalence of some chronic conditions” as well as the convergence of health, economic, and other inequities that “increase the risk of exposure, infection and death among Indigenous peoples,” including limited time to address health issues (especially for adults and elders), lack of health insurance, and lack of adequate care.^430^ As the additional health burden of these pre‐existing conditions combines with climate change, urban Indigenous communities (at least those in this study) face greater hardship than some other groups.^430^

Cultural survival and “biocultural work” by the Tuscarora Nation recognize traditional knowledge bases as critical resources for addressing climate change. The Grand Council of the Haudenosaunee offers programs that draw on and encourage sustained work with traditional teachings, including deepening understanding of traditional food bases, land stewardship, and efforts to support the Seventh Generation by “passing cultural knowledge and wisdom down to younger citizens.”^431^ The NIHB has an ongoing Climate Ready Tribes Initiative that focuses on funding Tribes to conduct local climate health work. Three Tribes have received those awards, although none in New York State have received them to date.^432^ However, NIHB does offer a Climate and Health Learning Community via its Climate Listserv.^432^

Recognizing that climate change affects Indigenous health indicators, several New York‐based Tribal Nations now have climate change adaptation plans in place. These include the Shinnecock and Saint Regis Mohawk documents described above, as well as climate action plans developed by the Tuscarora, Seneca, and Onondaga Nations. These plans emphasize that physical health and cultural health cannot be separated.^431^ These interrelationships for New York Indigenous Peoples are characterized as “cultural ecosystem services.” As traditional ecological knowledge and its impact on well‐being becomes better understood, the interrelationships between physical and cultural health become more valued. Figure 7‐5 illustrates some of these relationships in the form of a set of Indigenous health indicators developed by the Swinomish Tribe in Washington State. For example, the Swinomish Tribe consider stewardship of ecosystems, a key component of One Health, to be a health indicator equivalent to intergenerational teaching between elders and youth. This interconnected set of relationships recognizes the Tribe's understanding of, and advocacy for, a more holistic approach to climate change.

**FIGURE 7‐5:**
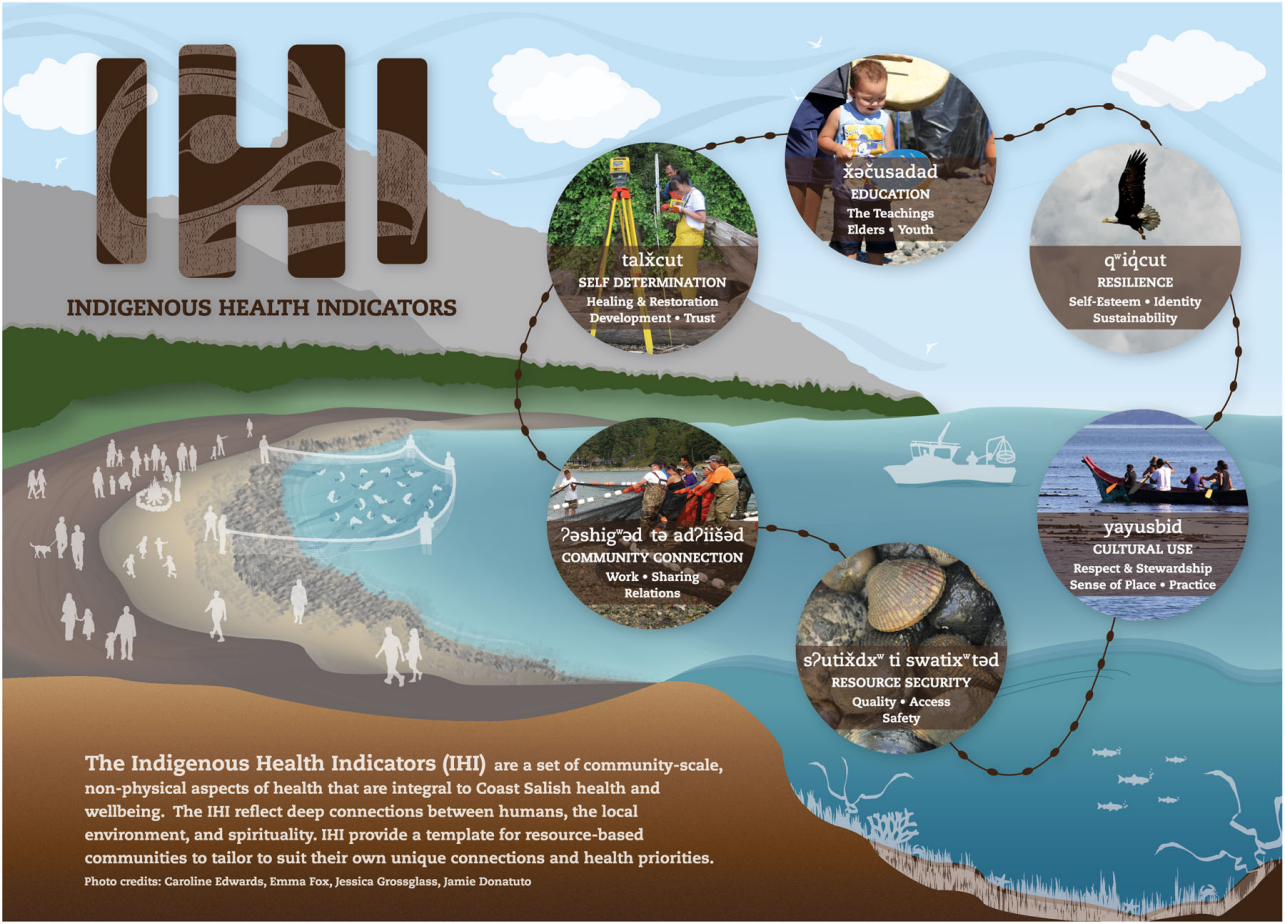
Indigenous health indicators from the Swinomish Tribe. This diagram shows examples of indigenous health indicators identified by the Swinomish Indian Tribal community in Washington state. Figure from Donatuto et al.^433^

### 4.3 Age

Age amplifies the negative impact of ambient temperature on health at both ends of the age spectrum. As adults age, their ability to thermoregulate decreases. Compared with their younger counterparts, older adults have a lower tolerance to heat stress from exercise and exhibit higher heart rates, lower stroke volume, lower cardiac output, higher mean skin and core temperatures, and lower sweat rates. Older adults are also at high risk of heat‐related morbidity and mortality because of their higher incidences of chronic disease, dehydration, and drug effects.^434^

At the other end of the age spectrum, children are physically more vulnerable to the effects of extreme heat, drought, and weather‐related disasters due to their size, physiology, and behavior (including spending more time outdoors). They are less heat tolerant than adults because their thermoregulatory systems are still developing. Young children cannot increase their cardiac output when faced with heat stress. While children have a greater ability to cool themselves by increasing blood flow to the skin than adults, their lower blood volume can cause heat‐related complications such as fainting and exhaustion. Children also have a lower sweat rate and sweat rate per body surface area than adults. Not only are children's sweat glands smaller, but they are also less sensitive to heat stress, which reduces their ability to lose heat from sweating in hot and humid environments. Children have a large surface area to body mass ratio, which increases their rate of heat absorption in hot environments because the absorption of solar radiation is spread over less tissue. Finally, because children's mobility and mental functioning are still developing, children may not be able to remove themselves from conditions that could induce heat stress.^434^, ^435^, ^436^

Learning is also compromised by the changing climate. Impediments to education can lead to lifelong health impacts such as chronic disease risk and shortened life expectancy. Elevated temperatures have been linked to poor educational outcomes among school‐age children. In a nationwide study of high school students, researchers revealed that without air conditioning, each 1°F increase in school year temperature reduced that year's learning by 1%, with hot school days especially affecting students of color, accounting for approximately 5% of the racial achievement gap.^437^ In a separate study of 4.5 million high school students in New York City, taking an exam on a 90°F day compared with a 72°F day was associated with a reduction in exam performance equivalent to one‐quarter of the Black–white achievement gap, which has long‐term financial implications for Black households.^438^ For further discussion on the educational impacts of climate change, refer to the Society and Economy chapter.

Children across New York State already bear a heavy burden of environment‐related disease from fossil fuel‐sourced air pollution and other toxics. Total costs of pediatric injury in the state are estimated at more than $4 billion per year, which “accounts for medical care costs, lost future wages, and quality of life.”^439^, ^440^ Moreover, children are more susceptible to indirect effects of climate change such as food shortages, conflict, negative economic impacts, and migration.^441^ Research on children who have chronic flood exposures demonstrates that children experience longer‐term impacts—particularly psychosocial impacts—than previously understood.^442^ Psychosocial impacts of climate change from direct and vicarious experiences, as well as long‐term developmental, educational, and economic consequences, make it harder for children to reach their full potential.^443^ Studies also suggest that the legacies of one generation manifest themselves in the next.^444^ Social inequalities such as educational and job attainment are transmitted over generations, as are depression and trauma, potentially affecting the psychological and behavioral development of the next generation.^443^ While integrated assessment models are widely used to evaluate climate policies over long periods of time and for different generations, intergenerational effects over time must also be considered in the context of climate‐related health policies.^445^ Children and future generations will disproportionately suffer the consequences of climate change.^443^

With extreme events such as heat waves projected to increase in frequency, intensity, duration, and extent, younger generations are expected to face more climate‐related adversity across their lifetimes than earlier generations.^446^ Extensive modeling studies estimated that under current climate pledges, children born in 2020 will experience a two‐ to sevenfold increase in extreme events, particularly heat waves, compared with people born in 1960.^446^ In 1987, the Brundtland Report described sustainable development in terms of intergenerational equity as “development that meets the needs of the present without compromising the ability of future generations to meet their own needs.”^447^ As of 2019, the world had 1.8 billion people between the ages of 15 and 29 years.^448^ Globally, the land area affected annually by heat waves was estimated to increase from about 15% around 2020 to about 22% by 2100 under a scenario limiting global warming to 2.7°F (1.5°C), and to about 46% under current emission reduction pledges. Though this global picture will vary across income and geography, future generations in the Global South are expected to be disproportionately burdened due to the combination of rapid population growth and level of exposure to extreme events.^446^ The study concluded that limiting global warming to 2.7°F (1.5°C) instead of following the current pledges scenario decreases the additional exposure of newborns to extreme heat waves by 40% and reduces the burden for wildfires by 11%, crop failures by 27%, droughts by 28%, tropical cyclones by 29%, and river floods by 34% globally, though it still leaves younger generations with unprecedented extreme event exposure.^446^

### 4.4 Occupation

Rising temperatures will increase the number of extreme heat days in the United States and have an impact on some workers. Outdoor workers, such as those in the construction, agriculture, and transportation industries, will be at greater risk due to both increased exposure to extreme heat and the amount of physical activity they exert under hot conditions.^449^ This concern was noted in New York State by the Attorney General and a coalition of delivery workers in their 2022 call for a federal heat standard for workers.^450^ Like outdoor workers, athletes who train and perform outside will also experience hotter conditions under a changing climate. Even indoor workers, such as those in warehouses, in structures under construction, or those near heat sources (e.g., bakers, cooks, welders) that are not air‐conditioned face a higher risk of heat‐related morbidity and mortality.^451^

Rising temperatures increase health risks for rural workers, such as farmers and agricultural workers (including migrant workers). Agricultural workers are more exposed to heat than the general population and may also be pressured to remain outside to meet production goals, increasing their risk. In addition, farm workers also may have greater exposure to pesticides as climate change increases pressure from weeds and insect pests, leading to increased use of pesticides.^452^

Industries in areas that will experience the highest changes in ambient air temperatures will suffer greater losses in labor. This will also affect the workers in these industries who are more likely to be socially and financially vulnerable.^395^ For example, the California Department of Public Health identified both agricultural workers and immigrants as some of the groups most vulnerable to climate change. New York State, like California and other agricultural states, relies on migrant farm workers. These workers not only experience climate inequity, but also face higher risks of fatal and non‐fatal work‐related injuries and more barriers to health care access.^453^

Outdoor workers such as forestry workers, veterinarians, military members, farm workers, and landscapers are also more likely to be exposed to vector‐borne illnesses such as Lyme disease.^454^ New York introduced a bill in 2022 (Senate Bill 8867) to “amend the workers compensation law to include Lyme disease and other tick‐borne diseases compensable, applying disability provisions that would apply to ‘disability caused by or in connection with’ such ailments.”^455^ As noted in Section 3.2.1.1, vulnerability to flooding is greater for those who work in emergency response and reconstruction, as many fatalities are associated with recovery activities.

### 4.5 Pre‐existing medical conditions

Chronic pre‐existing medical conditions sometimes amplify the impacts of various climate exposures, including exposure to extreme heat, extreme events, water‐related illnesses, and poor air quality.^456^ Several pre‐existing conditions place individuals at higher risk of heat‐related morbidity and mortality:

- **Respiratory illness**. Respiratory illnesses such as asthma and chronic obstructive pulmonary disease are exacerbated by exposures to aeroallergens such as pollen, as well as by exposures to mold, wildfire smoke, or other air pollution.
- **Cardiovascular illness**. Pre‐existing cardiovascular illnesses increase vulnerability to heat exposure and weaken an individual's ability to effectively manage climate‐related health issues, including mental health and stress.
- **Obesity**. Adipose tissue (body fat) has reduced thermal conductivity and an increased capacity to thermally insulate, reducing the body's ability to respond to increasing core temperature from heat stress. In addition to lower tissue conductance, obesity causes lower sweating rates, limiting total heat loss capacity.^457^
- **Diabetes mellitus (type 1 and type 2)**. Diabetes is associated with a reduced ability to thermoregulate.^458^ Diabetics can exhibit diabetic peripheral neuropathy (i.e., nerve damage), which can be associated with a partial or complete lack of sweating in some areas of the body.^458^ Blood flow to the skin, a known mechanism for lowering core body temperature, is delayed and reduced in individuals with type 2 diabetes.^84^
- **High blood pressure (i.e., hypertension)**. Individuals with hypertension exhibit higher cardiac work and skin temperatures than those with lower blood pressure.^459^ If the cardiovascular system is not running at optimal levels, the ability to thermoregulate suffers.
- **Pregnant people**. In pregnant women, the resting heart has been measured to be about 112 megajoules higher than before pregnancy.^460^ This means that those who are pregnant expend more energy at rest. Therefore, less energy is available for thermoregulation, putting pregnant people at higher risk of heat‐related morbidity and mortality.
- **Compromised immune systems**. Those with compromised immune systems may be more susceptible to heat, vector‐borne, or water‐related illnesses.^456^
- **Specialized care**. Those who need specialized care (such as consistent dialysis or mobility assistance) could suffer more in periods of service disruption and reduction or loss of access to care.^461^, ^462^, ^463^, ^464^, ^465^

### 4.6 Mental and behavioral health conditions

There is a well‐documented connection between increased vulnerability to extreme heat and mental or behavioral health disorders.^72^, ^466^, ^467^ This increased risk is attributed to multiple factors, including the effects of medications, the inherent physiological response to substances such as opiates and alcohol, and the cognitive effects of substance abuse or misuse and associated inability to take protective action against extreme heat.^466^ A study examining the relationship between mental illness hospitalizations and temperature in New York State found that from 2009 to 2016, emergency room visits for mental disorders were most frequently related to psychoactive substance abuse.^468^ For chronically exposed populations and those with extreme flooding experiences, mental health also remains at risk.^186^, ^469^

Given that future temperatures in New York are expected to rise, a variety of mental health services will likely be needed to assist this at‐risk population.

### 4.7 Housing situation

Where people live affects their risk of climate‐related health and safety impacts. Equitable housing and the associated costs of healthy and safe shelter are a critical issue, as populations with health and social vulnerabilities are disproportionately affected by climate concerns at home.^470^ For example, New York State has the largest public housing stock and resident population in the country. As a result of major capital backlogs, high poverty levels, and other barriers, public housing residents contend more with the impacts of extreme weather (including flooding and high heat) than do other populations. These amplified health risks were evident during Superstorm Sandy.^471^ Housing type, age, tenure, and other factors are important considerations when assessing climate risks.^270^

Individuals residing in institutions that provide care (e.g., hospitals, nursing homes, adult‐care facilities, primary and mental health care facilities) often have existing health conditions and are older, bedbound, or lacking in mobility, all of which are risk factors for heat‐related morbidity and mortality.^472^, ^473^, ^474^, ^475^ Individuals residing in incarceration settings experience multiple risk factors that increase their thermal vulnerability, including social isolation, disproportionate mental health issues, comorbidities, limited mobility, and a reliance on external factors to provide a safe, healthy environment.^476^ Further, there may be increased heat exposure in older incarceration facilities due to poor ventilation and overcrowded conditions.^477^ As of 2015, New York was one of 22 states that lacked policies on temperature regulation in prison facilities.^477^

Individuals experiencing homelessness, particularly those who reside outdoors, face an elevated risk of heat‐related medical conditions. The New York City Department of Homeless Services’ annual report on mortality rates of people experiencing homelessness notes that excessive heat and cold are among the causes of death due to external factors.^478^ Characteristics that are more common among individuals experiencing homelessness (i.e., pre‐existing psychiatric illness, cardiovascular disease, pulmonary disease, advanced age, living alone, being socially isolated, not having access to air conditioning, alcoholism, tranquilizer use, cognitive impairment) are known risk factors for death during heat waves. Furthermore, unhoused individuals who spend time outdoors in urban areas are exposed to elevated temperatures due to the urban heat island effect.^479^

### 4.8 Geography

Rural New Yorkers—previously thought to be buffered from extreme hot weather because they reside outside of urban heat islands—actually show a risk of heat‐related morbidity and mortality similar to that of urban New Yorkers, and their risk starts to increase at lower temperatures.^73^ As temperatures rise with climate change, these individuals may be more at risk, especially if they live in older houses that lack air conditioning. The Buildings chapter discusses the vulnerability of manufactured housing in extreme weather events.

Communities located in coastal areas are vulnerable to storm surges, which are projected to become more frequent and intense because of climate change. Storm surge and flooding threaten coastal populations with potential displacement and health impacts. As with inland flooding, public health and safety are threatened by waterborne pathogens, poor air quality, drowning, hypothermia, electrocution, and chronic mental health consequences. In coastal areas that are heavily urbanized, such as New York City, the density and degree of impermeable surfaces creates unique risks of urban heat islands and pluvial flooding. The concentration of population in these areas amplifies exposure impacts and creates greater burdens on municipal resources.^480^

Climate change impacts affecting the Great Lakes region include increased flooding as storms and cloudburst events become more pronounced, more invasive species due to warming lake waters, more HABs, less habitat, more toxic chemical exposure from leaching, contaminated runoff (e.g., *E. coli*, industrial products) exacerbated by extreme precipitation, lake‐effect snow, changing amounts and frequency of freezing rain, and more infrastructure failures (such as crumbling shorelines due to intensifying storms).^481^, ^482^, ^483^ Research on social vulnerabilities in the Great Lakes region identifies levels of high vulnerability in the Ontario Basin in New York State. Out of the areas studied, the Ontario Basin had the second‐highest socioeconomic vulnerability and the highest housing type and transportation vulnerability.^484^ To address climate‐driven disturbances and community response, the Climate Governance Variability in the Great Lakes Research Coordination Network was recently established.^484^, ^485^

### 4.9 Access to air conditioning

Reliable air conditioning is a recognized preventative measure for indoor heat exposure, yet not all New Yorkers have it. Access to cooling is inequitably distributed and remains inaccessible or unaffordable to many vulnerable populations in the state. Even residents who have air conditioning systems might operate their systems sparingly due to utility cost concerns or the poor performance of older units.

A 2021 study of equitable access to cooling in New York City found that almost 300,000 housing units across the city, or approximately 750,000 residents (assuming an average household size of 2.6 residents per home) did not have air conditioning.^486^ New York City residents are less likely to have air conditioning if they live in older homes or if they have low incomes or live in poverty.^391^, ^487^ Older homes could also have poorer quality weatherization, and therefore, greater exposure to temperature extremes.

A 2015 survey found a strong association between air conditioning access and race/ethnicity in New York City. Researchers found that the odds of an adult not possessing in‐home air conditioning were considerably greater for non‐Hispanic Blacks compared with other racial and ethnic groups.^137^ Inequitably distributed access to air conditioning partially explains disparities in observed heat‐related health outcomes.

Lower rates of air conditioning access and higher percentages of multiple measures of poor housing conditions at the neighborhood level have significant positive associations with heat‐related death rates in New York City.^488^ The New York City Department of Health and Mental Hygiene reviewed the 48 heat‐related deaths in New York City after heat waves in 2008–2011 and found that 85% of the deaths occurred at home, and none of those who died had working air conditioning.^489^

Another consideration is that not all air conditioning systems are equal in mitigating heat. In a study of New York City apartments, residences with central and ductless air conditioning types had notably cooler temperatures than those with window and portable air conditioning units. Further, top‐floor apartments had much higher temperatures than lower‐floor apartments.^490^

Fewer data are available regarding air conditioning penetration and access to cooling in New York State outside of New York City. It is worth noting that because extreme heat is projected to increase across the state, including parts of New York that historically have not experienced extreme heat on a regular basis, access to cooling will be increasingly important for vulnerable populations throughout New York State.

### 4.10 Access to care

Climate‐sensitive health threats pose particular risk to people who lack reliable access to affordable health care, and climate change itself disrupts the operation of health systems, which reduces equitable access to health care; people with chronic health conditions are particularly vulnerable.^42^, ^43^ Climate hazards in New York State, including extreme heat and extreme precipitation, can disrupt power to health care providers’ buildings, inhibit health care providers from reaching work, and endanger physical health care infrastructure.^43^

Power disruptions can also impede in‐home care and at‐home medical devices and prevent people from going to the hospital. For example, power outages during Superstorm Sandy affected multiple hospitals, resulting in disrupted care for hospitalized patients, including people receiving dialysis treatment.^491^, ^492^, ^493^ Flooding also impeded emergency medical service access to homes and access to hospitals and other care settings. A recent workshop from Health Care Without Harm raised the issues of personnel unable to get to work, risks to supplies (undamaged, available, and deliverable), and interruptions in utilities (power, water, gas, sewage, waste disposal).^494^ Moreover, as Sandy recovery proceeded, New York City hospitals recognized the need to not only shore up their facilities, but also to revisit their emergency response plans.^495^ In 2006, flooding in Binghamton forced Our Lady of Lourdes Hospital to evacuate patients and shut down operations for more than a week, affecting critical care to people in the area (refer to the Hospital Floodwall Retrofit case study for more information). Reciprocally, during flood events, patients are less likely to keep appointments, resulting in missed care, delayed care, and potential worsening of chronic conditions or the development of new health problems. Extreme weather events can also disrupt patients’ medication deliveries or their ability to access pharmacies.^496^, ^497^

Many rural areas of the state already lack sufficient access to health care and are classified as medically underserved or a health professional shortage area by the U.S. Health Resources and Services Administration.^498^ Some rural counties in New York have no hospitals, while other facilities have reduced services in recent years. Rural populations who already have limited health care access will be especially vulnerable to extreme events that could close already‐scarce facilities or make transportation to those facilities more difficult or impossible.

Disruption of infrastructure and services can have long‐term effects on the use of services in affected areas, especially among vulnerable populations.^45^ For people with disabilities and dependence on support programs, there is even greater stress in accessing care.^41^ Access to care is an ongoing challenge for these individuals; climate events amplify these challenges via health service system disruption, transportation disruption, power outages, hospital evacuations or facility damage, or displacement of caregivers.^44^

## 5 CASCADING, COMPOUNDING, AND CROSS‐CUTTING ISSUES

Climate change acts across a range of human‐environment systems, creating impacts and risks that are increasingly complex and difficult to predict and manage. Though health outcomes are typically assessed individually, effects often do not occur in isolation. Exposures cascade (e.g., extreme heat, power outage, medical device stops working) and exposures compound (e.g., a hurricane disrupts power during an extremely hot period).^499^

Cascading exposures highlight the interdependencies of systems.^500^ For example, heat waves and drought are physically connected by feedback loops that can amplify drying and heating, causing prolonged hot and dry conditions that eventually negatively affect food security and ecosystem resilience.^501^

Comparatively, compounding events may be largely unrelated to each other, but their co‐occurrence can exacerbate the impacts of both. Superstorm Sandy offers one example. In October 2012, Sandy made landfall in New York and New Jersey. A nor'easter struck a week later. The combination of storms in the region caused flooding and power outages at refineries, pipelines, and petroleum terminals that affected 8.7 million customers and led to stock drawdowns, temporary price increases, and health impacts.^502^, ^503^

Conditions do not need to be extreme, but instead co‐occurring and interacting, to generate such threats.^501^ For example, the cascading impacts of heat waves contribute to drought conditions, which can compound with energy insecurity and power outages to overtly threaten those with fewer resources (e.g., alternative housing, ability to pay, additional power support such as a generator).^501^ Impacts will emerge across different places, times, and timescales, and multiple impacts will occur simultaneously and in different combinations. Additional research into the co‐occurrence of these multiple threats could improve the understanding of both the scope and scale of these concerns, as the extent to which these impacts could cascade and compound to form multiple impacts across sectors is not yet fully understood.^500^

### 5.1 Connections to other chapters

Climate hazards such as extreme weather events, which sometimes occur in rapid succession, can disrupt health care, transportation, energy, finance, and other systems in ways that can have acute and chronic impacts on health.^504^ Systems at risk covered in other chapters of this assessment include buildings, water, transportation, energy, ecosystems, and society and economy. Other chapters explore the specifics of those systems, while the findings below examine the health implications when such systems are at risk from increased temperatures, increased precipitation, more extreme storms, and sea level rise. Within each system, the potential for cascading and/or compounding events is relevant.

#### 5.1.1 Agriculture and food systems

Climate change is expected to affect food production and supply by decreasing availability, access, quality, and stability of food systems.^353^, ^452^ Access to refrigeration along the food supply chain under the changing climate is an important consideration, as revealed during the COVID‐19 pandemic.^505^, ^506^

Food systems also include Indigenous communities’ foraging and harvesting of culturally relevant plants, shellfish, and animals. This food system will also be disrupted by shifting climatic zones attributed to climate change. The wild strawberry plant, for example, has nutritional and medicinal qualities, and its availability has already been limited.^421^

Various sectors of the population will be affected according to their ability to respond to vulnerabilities and disruptions. Climate‐fueled impacts could create food insecurity and harm health disproportionately for some populations. Human health depends on quality nutrients being available in food, which could be compromised by rising carbon dioxide concentrations, changing precipitation patterns, changing temperature, and lack of sufficient water in the places where the food is grown.^353^ Food insecurity is associated with an increased risk of birth defects, anemia, lower nutritional intakes, cognitive problems, aggression, and anxiety.^507^

#### 5.1.2 Buildings

Many buildings lack weatherization strategies to maintain comfortable occupancy as temperatures rise, which increases occupant health exposures to weather extremes. Other buildings are at risk from pluvial or fluvial flooding, or from coastal storms. With primary shelter at risk, New Yorkers could stay in their homes and address such risks or evacuate to other locations, such as others’ homes, community shelters, or hotels. However, few New York municipalities have neighborhood‐level shelters aside from school buildings. Also, many families lack alternatives to remaining at home.

Centralized shelters frequently introduce new challenges, such as how to deal with pets, manage density during a pandemic, and ensure personal safety.^508^ Given limited availability, capacity, and personal comfort, community members sometimes remain in their homes, even when those homes are at risk, in lieu of evacuating. Those with fewer resources could be unable to evacuate to hotels.

As evidenced from Superstorm Sandy and Hurricane Ida, New York's aging building stock coupled with increased storm intensities and larger cloudburst events will increase risks to public health as people seek to shelter in areas prone to flooding, including basement apartments and buildings constructed in floodplains that lack adaptation measures.^198^ Similarly, during heat waves, New Yorkers could choose to remain in overheated homes or (for those unhoused) remain outdoors, even as temperatures reach unhealthy levels.

Compounding this problem is the increased exposure of health service facilities to climate change impacts. For example, critical facilities in flood‐prone areas might not be able to accept patients or serve emergency response needs.^509^ While substantial investments were made following Superstorm Sandy, climate adaptation programs continue to require investments to manage ongoing change. As recently as 2022, the U.S. Department of Health and Human Services asked health systems to commit to further climate action, including readying facilities for greater resilience.^510^ NYC Health + Hospitals signed this commitment in May 2022. Today, for NYSDOH‐regulated facilities, NYSDOH approves plans for construction at hospitals and nursing homes and regulates their operations. These facilities represent critical services and operations, and thus have a critical need to plan for climate impacts.^270^

#### 5.1.3 Ecosystems

Increased temperatures and increased flooding will further degrade ecosystems (i.e., parks, beaches, forests, rivers, lakes, and other respite areas), making them less safe for community members even as their importance for personal respite increases.^511^, ^512^

Evidence suggests that rising temperatures, recurrent floods, or saltwater intrusion could cause plants and animals to no longer be able to tolerate the physical conditions of their historical range, resulting in local extinctions and/or species migration.^513^, ^514^, ^515^ The loss of local species can harm the mental health of residents who have a personal connection to those species. For communities such as Tribal Nations with particularly strong connections to land and ecosystems, these changes could have an even greater impact on well‐being.^282^ When investing in green infrastructure for climate adaptation, municipalities must consider which plant species are likely to thrive in a warming environment.^114^, ^513^

#### 5.1.4 Energy

Energy insecurity is defined as the lack of adequate energy in the home. It is a multidimensional phenomenon that takes into account the physical, behavioral, and economic conditions of a household.^516^ Energy insecurity is a central criterion directly related to increased health risks from climate change, particularly in terms of the costs and the access to heating and cooling in homes. In addition to the chronic issue of energy insecurity, power outages—driven by both extreme storms and extreme heat—are also a potential important health risk factor.^517^ Following Superstorm Sandy, carbon monoxide exposures increased in the New York City area, mostly related to indoor grilling and improper generator use.^518^, ^519^

Increases in extreme weather events mean more need for power in extreme heat or extreme cold. Extremely hot or cold days mean households are running air conditioners or heaters to stay safe, which strains power systems. In periods of extreme cold, households that do not control their heat might rely on space heaters, which can pose a major fire risk, or they might use ovens for heat, which raises the risk of potentially fatal carbon monoxide poisoning. Extreme heat days can lead to power outages or brownouts, leaving customers without adequate energy. Lack of adequate energy can harm health and well‐being, particularly for those relying on air conditioning (or electric heating in winters) to maintain comfortable temperatures. People with chronic illnesses, such as chronic obstructive pulmonary disease, also rely on electricity for life‐saving medical devices. In an extreme weather event and its aftermath, lack of energy to power devices can be a notable contributor to death or hospitalization. Power outages can have a range of impacts, such as spoiling food or hindering Americans with Disabilities Act‐compliant access when elevators stop working.^520^ Health care facilities, communities, and individuals need contingency plans when there is a disaster and potential disruption in utility service.^521^ To meet a shifting need for energy on increasing extreme temperature days, a more resilient power system that includes renewables such as solar and wind energy, and homes that use energy more efficiently, can reduce strain.

Energy‐related financial costs are stressors that can inhibit vulnerable populations from accessing and using lifesaving air conditioning (refer to Section 4.9). Research in New York City has documented uneven distribution of air conditioning‐related economic burdens, with low‐income neighborhoods experiencing the largest relative costs.^522^ Lack or nonuse of an air conditioner was cited in 100% of heat‐related deaths with detailed records in New York City from 2011 to 2020.^75^ However, interventions to change traditional temperature set points, weatherize, and install reflective roofs can help reduce the household energy burden.^523^

#### 5.1.5 Society and economy

Climate change will affect most aspects of New York State's economy and social fabric, challenging longstanding cultural traditions and affecting more vulnerable regions and populations disproportionately. Cultural ties and the resilience that stems from social cohesion will be disrupted with increasing precipitation and storm surge events, which in some cases could lead to wholesale relocation of communities. With inequities in rural and urban areas, these disruptions will unequally impact New Yorkers, exacerbating pre‐existing conditions and worsening already disparate health outcomes.^281^

#### 5.1.6 Transportation

Increased precipitation that interrupts safe transportation^524^, ^525^ will in turn directly and indirectly affect health. For example, interruptions, delays, and halts to transit interfere with access to health appointments, which is particularly problematic for those with underlying conditions requiring consistent care, such as dialysis. Public transit access is also recognized as a critical resource for those trying to access New York State cooling centers.^136^ Increased temperatures for riders, whether on buses, at stations, or on subways, will also affect public health.^526^

Major storms such as Superstorm Sandy can introduce long‐term public transit interruptions as well as fuel supply limitations, reducing the capacity of private vehicles as alternate support. Relatedly, an 18‐ to 283‐fold increase in gasoline exposure—mostly associated with siphoning due to shortages—was observed in the wake of Sandy, with a corresponding risk of gastrointestinal and respiratory symptoms. While most “exposures were managed at home with minimum clinical toxicity, some patients experienced more severe symptoms.”^527^, ^528^

#### 5.1.7 Water resources

Extreme precipitation events do not always happen in isolation. For example, New York State experienced snow and freezing temperatures 8 days after Superstorm Sandy, which delayed recovery, stressed a fragile infrastructure, and put more people at risk for negative health outcomes through carbon monoxide exposure from generators.^518^ Winter storms in the state, especially when accompanied by power outages, have also been linked to hospital admissions for waterborne and foodborne illness, as well as other impacts.^529^ Concurrent and cascading impacts can exacerbate community vulnerabilities. For example, increased precipitation will further expose community members to floodwaters; contaminate recreational areas, reducing their use; and damage ecosystems.^114^, ^530^ Rising sea levels will raise groundwater in coastal areas, affecting infiltration and functioning of septic systems. Seawater could also contaminate aquifers providing well water in coastal zones.^115^ Warmer waters further expose people to bacteria, such as *Legionella*, and cyanobacterial algal blooms.^531^ Lengthy recovery periods following major events have the potential to affect New Yorkers in the near term as the event and its aftermath experiences unfold, and in the long term as compounding consequences amplify the event, particularly for those most vulnerable.

### 5.2 Public health infrastructure engagement

New York State's health care and public health infrastructure has a large role to play in promoting and implementing climate adaptation and resilience. Yet, public health engagement requires knowledge of local climate hazards, vulnerability, adaptive capacity, and coordination to protect public health. In New York, the involvement of public health professionals in climate change planning is evolving. New York's 2019 Climate Leadership and Community Protection Act (Climate Act) calls for health department staff to assist in formulating the scoping and climate action plans, as well as in defining criteria for “disadvantaged” communities as defined in the Act.

NYSDOH and the New York State Association of County Health Officials (NYSACHO) hosted a workshop series to raise awareness, build a community of practice, and provide a forum for local health departments and other local partners to discuss plans to address key climate and health priorities in recognition of the importance of local planning and adaptation work. Multiple training opportunities for clinicians on climate and health now exist as well.^532^, ^533^, ^534^

A 2022 study assessed the role of public health in the adaptation plans of 22 large cities worldwide that are recognized as highly health‐adaptive.^535^ It found that 90% of the cities had actions planned for at least three of five health‐associated adaptation activities, but only 73% involved a public health agency, even as other agencies had a notable presence in the plans.^535^ The Association of State and Territorial Health Officials surveyed health agencies in 2020 and 2021 to better understand how agencies were planning to address climate change and extreme weather.^536^ A key finding was that most of the respondents “do not regularly evaluate their capacity to address climate change and health.”

To build climate change adaptation capacity in the public health community, the CDC devised Building Resilience Against Climate Effects (BRACE), a comprehensive framework for developing local climate change adaptation plans. The framework offers five steps to provide an iterative approach to adaptively manage the health effects of climate change:

1. Forecast climate impacts and assess vulnerabilities
2. Project the disease burden
3. Assess public health interventions
4. Develop and implement a climate and health adaptation plan
5. Evaluate impact and improve the quality of activities

The framework assesses the most concerning public health risks in a region, acknowledging the place specificity of emerging climate‐related threats to public health.^537^ NYSDOH used this framework to develop a climate and health profile in 2015 to help public health professionals in the state with adaptation planning, implementation, and evaluation.^403^

The BRACE framework emphasizes the importance of greater integration of health and climate change. For example, the statewide extreme heat action plan presently under development will offer a powerful tool to engage all state health departments, health service providers, and associated health care professionals in raising risk awareness, identifying locally relevant coping resources, aiding in hazard mitigation strategy development, and building toward long‐term adaptation.^538^ With a mandate in place, and resources being gathered, this planning process could offer new ways of engaging communities across the state to improve near and long‐term climate‐related health outcomes.

Oregon is requiring Medicaid‐managed plans to cover certain climate adaptation expenses.^539^ The process is complex, and states implement these interventions under federal waiver programs, usually with state plan amendments. While New York State does not yet have as overt and specific a program as Oregon, it has implemented programs such as using federal low‐income home energy assistance program funds to pay for air conditioners for at‐risk populations under a federal block grant program administered by the Office of Temporary and Disability Assistance.^540^ One of the Extreme Heat Action Plan Work Group's interim recommendations is to assess “existing resources and capacities and proactively identify and secure needed new funding to ensure effective implementation of recommended short‐ and long‐term actions”; “detail funding sources and commitments as well as implementation timelines for each action contained in the adaptation plan”; and “work with the State legislature, federal partners, and other funding sources to dedicate adequate funding for implementation.”^541^ To do so effectively, increasing public health support, creatively leveraging related programs such as the Home Energy Assistance Program, and developing a system of interconnected efforts centered on well‐being will be crucial to sustained engagement and successful implementation. As Fox et al. argue, “public health has an essential role in climate planning and action,” but their research shows that public health response to climate change “has been promising in the area of assessment (monitoring climate hazards, diagnosing health status, assessing vulnerability); mixed in the area of policy development (mobilizing partnerships, mitigation and adaptation activities); and relatively weak in assurance (communication, workforce development and evaluation).”^542^

### 5.3 Community outreach and engagement

Community involvement and adequate support resources are broadly recognized as necessary for effective adaptation and planning. To be involved, communities require “(1) multistakeholder engagement, (2) cross sector (pertaining to more than one group or department) vulnerability assessment and strategy development, and (3) an iterative cycle of learning and improvement over time.”^543^ This is termed “whole community resilience” or a “whole of community” approach.^543^, ^544^, ^545^ For policies to be most effective, communities need opportunities to share their knowledge and concerns, particularly as they relate to other chronic conditions tied to housing and economic stability, with policymakers. Public health teams play an important role in the development of messaging that connects climate change, vulnerability, and health outcomes. Policymakers in turn can prioritize processes that recognize and integrate diverse representation for local knowledge, history, and the lived experience and that build from existing community work and leadership.^546^

NYSDEC developed the Climate Smart Communities Program (CSCP), a framework and accompanying guidance for communities to take stakeholder‐driven action toward local greenhouse gas mitigation and climate adaptation, including actions to reduce health risks.^547^ Action begins with the creation of a task force of residents, local officials, professionals, and stakeholders who are knowledgeable about the local decision‐making process and how climate change will affect the quality of life, thereby leveraging local, traditional, and scientific knowledge bases. The task force meets regularly to collaboratively plan, implement, and track local climate efforts choosing from a broad range of suggested science‐based actions, allowing for local collaboration, planning, and prioritization. NYSDOH staff have worked to integrate public health into CSCP certification actions.

As of December 2023, 399 communities across New York State are participating in CSCP, representing approximately 9.5 million residents (Figure 7‐6). The CSCP has yielded such tools as the Hudson River Flood Impact Decision Support System,^548^ an interactive science‐based tool for stakeholder decision‐making developed by Columbia University; the Community Climate Adaptation mapping tool developed by Westchester County Geographic Information Systems^549^ to aid in community engagement, planning, and communication; and New York City's Climate and Health Hub^15^ to help facilitate local planning processes. As previously mentioned, NYSDOH has also developed several resources around the health consequences of climate effects, such as the Extreme Heat and Health StoryMap, the Heat Vulnerability Index, and the County Heat and Health Profile Reports for local health departments and the public.^131^, ^133^, ^550^, ^551^

**FIGURE 7‐6:**
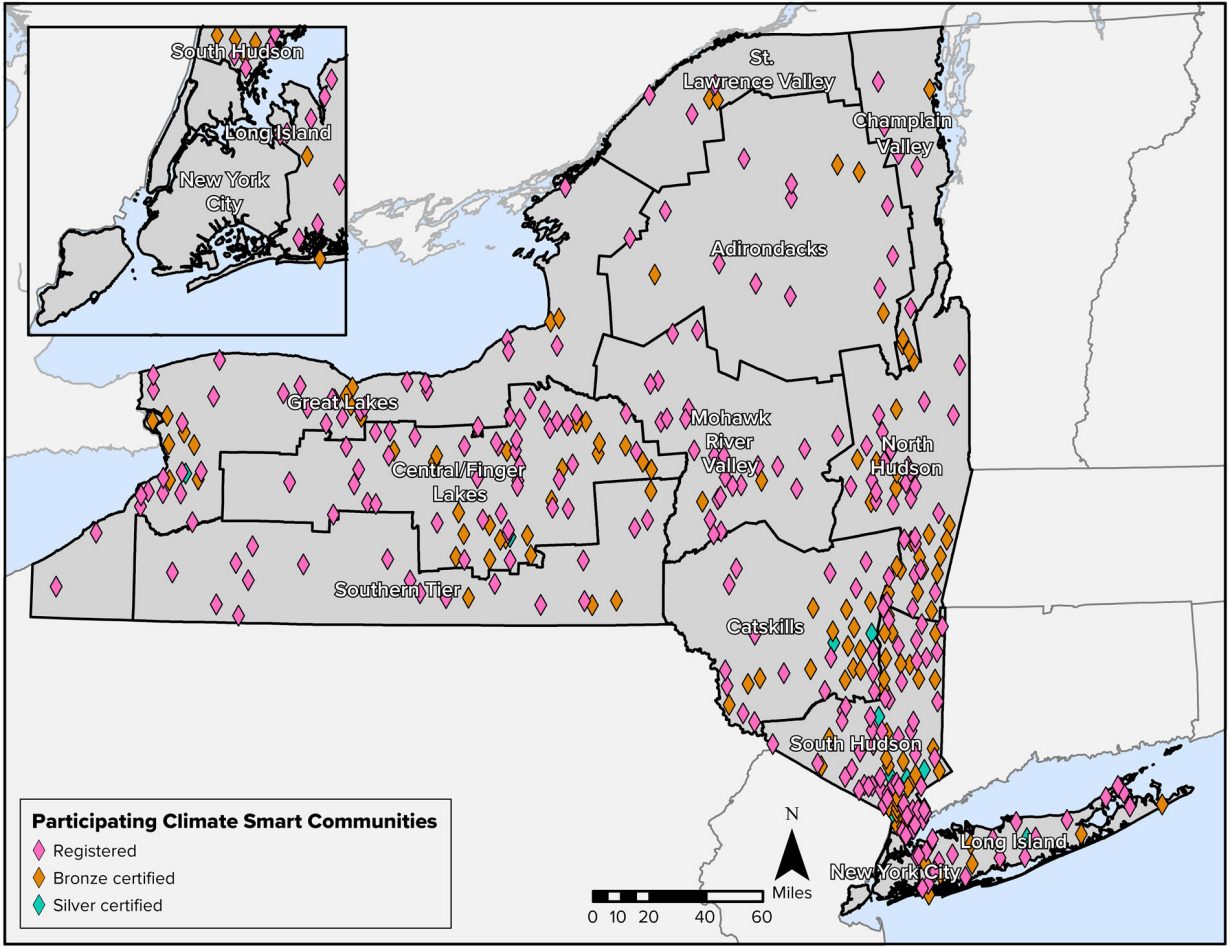
Climate Smart Communities participants as of December 2023. Data from New York State Department of Environmental Conservation (2023).^560^ The latest data can be viewed online at the New York State Climate Smart Communities website.^547^

The majority of New Yorkers—particularly environmental justice communities that bear the brunt of climate impacts—understand that climate change is happening and that it is driven by human activity.^552^ However, only about a quarter of New Yorkers think climate change is very important to them personally.^552^ Frequently, messaging around climate change does not make the connection between climate change and the everyday stressors that people are navigating, such as housing, jobs, or other aspects of daily life.^553^, ^554^ Health impacts of climate change are an important framing that can help people understand the gravity of the topic.^555^ As one example of such framing, Erie County initiated a community engagement process in 2021 that solicited input on developing an equitable climate action plan for a healthy and resilient county.^556^, ^557^

Additional public engagement on climate and health can raise awareness and link communities to resources for near‐term coping while hazard mitigation and adaptation investments are underway. Not all communities are engaged yet in climate action. As Figure 7‐6 shows, participation in the CSCP is more prevalent in the more urbanized portions of New York State. With the development of the 2023 State Hazard Mitigation Plan, which includes developing local details,^558^ New York has a prime opportunity to add public health to ongoing discussions. New York is involved in careful consideration and mapping of climate‐related health and other impacts. To that end, the New York Division of Homeland Security and Emergency Services is using indices, such as the Centers for Disease Control and Prevention's Social Vulnerability Index, to identify local health risks (Figure 7‐7).^559^

**FIGURE 7‐7:**
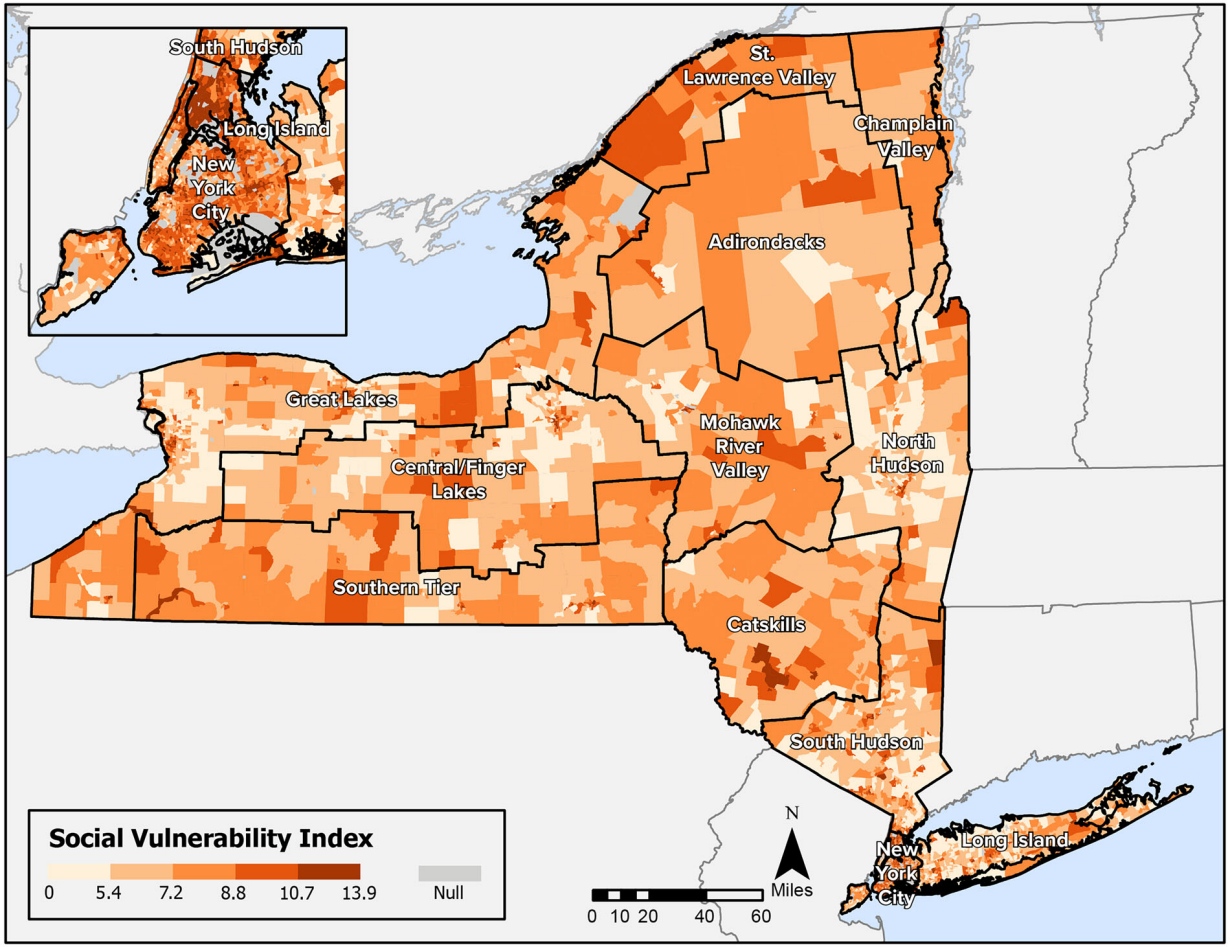
Centers for Disease Control and Prevention's Social Vulnerability Index (CDC SVI) by census tract. A higher SVI score indicates “communities that may need support before, during, or after disasters.” Figure adapted from New York State Division of Homeland Security and Emergency Services (n.d.)^559^ and Agency for Toxic Substances and Disease Registry (2024).^561^

### 5.4 Climate change and pandemics

Climate hazards such as heat waves and floods can pose a double health risk during a pandemic, such as COVID‐19. As the climate changes, populations (flora and fauna) shift to escape risks, concentrating in some cases and creating new interactions between populations in others. These shifts in population dynamics may in turn increase the likelihood of virus emergence. How climate change will impact the spread of zoonoses (diseases that are transmitted between humans and animals) remains uncertain.^562^, ^563^ Furthermore, the relationships between pathogen transmission and contributors to climate change, such as deforestation and greenhouse gas emissions, warrant further research.^564^

Given growing concerns about novel agents and viral spillover from melting glaciers,^565^, ^566^, ^567^ pandemic planning must be further integrated into climate‐related public health services, such as increasing ventilation/filtration and establishing proper protocols to prevent the spread of infectious diseases (e.g., COVID, monkeypox, polio) in cooling and evacuation centers.^568^, ^569^ In 2021, the CDC specifically called for the integration of One Health to better address the relationships between public health, other species, and our environments, describing it as a “crucial” approach to address new diseases.^570^

BOX 2 **Climate change and COVID‐19**

Climate change and COVID‐19 are environmental justice issues: elevated social vulnerability, COVID‐19 infection rates, and extreme heat exposure occur simultaneously in certain communities.^522^ There is no conclusive evidence that ambient temperatures—now and in the future—have a strong influence on COVID‐19 transmission,^571^, ^572^ yet the COVID‐19 pandemic together with extreme heat resulted in multiple health risks, with low‐income communities and communities of color experiencing an inordinate amount of the health burden due to ongoing impacts of structural racism.^35^, ^410^ Older adults and individuals with pre‐existing health conditions experience magnified health disparities and disproportionate suffering. In New York specifically, the pandemic response added challenges for people who already experience increased risk from climate hazards due to baseline health and their social group.

The COVID‐19 pandemic also revealed limitations within public and private health systems, which simultaneously had to cope with escalating climate challenges, the pandemic itself, and the diversion of resources from other public health services.^573^ While multiple states have resources to integrate climate change into local health department programs,^574^, ^575^ and while New York adopted the ambitious Climate Act in 2019,^576^ public health infrastructure still lacked the structural complexity required to tackle these compounding events.^577^ Even when resources were available, many local departments were overwhelmed with immediate COVID‐19 response and did not have adequate capacity to manage resources.

The risk of COVID‐19 transmission was found to be greater indoors than outdoors,^578^ which was a concern for those using indoor spaces for refuge from high temperatures during the pandemic. Thus, some disaster response protocols—such as using public cooling centers and shelters—conflicted with COVID‐19 guidelines of sheltering in place and social distancing.^579^ Staying at home could increase the risk of heat‐related illness due to the low availability of air conditioning and its energy cost burden, although recent Home Energy Assistance Program reforms intend to address that challenge.^580^ Going to a cooling center could increase individuals’ risk of COVID‐19 due to close contact with others. In addition, while public transit vehicles can serve as refuge and service for essential mobility and evacuation during heat waves,^581^ these typically enclosed, indoor‐like spaces—as well as artificial shade structures and trees at transit stops and stations—place individuals in proximity to others, potentially increasing the risk of viral transmission. In 2020, New York City quickly rolled out a COVID‐19 Heat Wave Plan, which led to a rethinking of approaches to provide cool spaces while shelters closed due to COVID‐19‐related limitations on gathering sizes.^582^, ^583^

## 6 LOOKING AHEAD

This section looks at opportunities for positive change that can grow out of climate adaptation efforts and identifies emerging topics and research needs in human health and safety. The section concludes by summarizing the major findings and recommendations presented in the chapter.

### 6.1 Opportunities for positive change

Responding to climate change impacts in a thoughtful, inclusive manner could provide opportunities to improve health and safety outcomes for New Yorkers. For example, one study estimated that rising temperatures with climate change would result in a net increase in bike usage in New York City.^126^ Another found that climate change could lead to a net increase in physical activity participation across the state.^127^

On a broader scale, strategies to reduce greenhouse gas emissions and build climate resilience frequently have multiple immediate health benefits.^49^, ^50^ The health benefits of reducing greenhouse gases (called cobenefits due to simultaneously improving air quality while also addressing the root cause of climate change) have been the focus of extensive research.^584^ These benefits—estimated at billions of dollars annually in health benefits for New York City alone under some pollution reduction^585^ strategies—come primarily from reducing the combustion of fossil fuels (coal, oil, gas) from buildings and vehicles.

Moving away from localized sources of pollution, such as oil heating in buildings and diesel fuel in trucks, is an important step in attaining benefits, but transitioning electricity‐generating power plants to less‐polluting fuel sources is even more critical as electricity demand grows.^523^ Air quality improvements due to the Regional Greenhouse Gas Initiative in the Northeast were estimated to have delivered substantial benefits to children's health alone, saving $191–$350 million over the 2009–2014 period.^586^ Child health benefits in New York City from a proposed regional transportation and climate initiative were anticipated to eventually save a projected $22 million per year in health‐related costs and bring a higher proportion of those benefits to communities of color, who currently experience the highest burden of environment‐related diseases.^587^

Examples of proactive measures that concurrently address health, safety, and climate resilience include:

- Using climate hazard mitigation and adaptation actions to raise awareness of health risks, offer near‐term coping resources, develop hazard mitigation strategies related to health outcomes, and frame adaptation strategies (e.g., train health professionals, monitor health indicators, track public awareness of risks) in a “whole of community” approach.
- Providing local health departments with resources to develop locally relevant content and offer outreach programs.
- Broadening engagement with New York State health service providers to encourage climate change and (physical and mental) health discussions with patients to raise risk awareness, introduce coping resources such as energy assistance or home weatherization programs, and share best practices on how to manage extreme heat impacts to mental health medications.
- Reducing hazard impacts on associated chronic and acute mental stresses by providing guidance on adaptations to growing health risks, assuaging anxieties, and offering hope as well as applied solutions.
- Syncing climate and health programs with chronic disease management and prevention programs and other efforts to support people with climate‐sensitive vulnerabilities.
- Quantifying costs of climate change–related health burdens to the health system and including those costs in benefit‐cost analyses to prioritize capital and operational investments to curb climate change impacts.
- Addressing historical racist policies (structural racism) and their long‐term negative health outcomes. This includes ensuring that climate change strategies include plans and resources to address equitable preparation, hazard mitigation, and adaptation.
- Establishing, planning, and budgeting for programmatic strategies to enable pandemic preparedness and response to coexist with climate change preparedness and response.
- Promoting climate adaptation and resilience through certificates of need, licensure, federal block grants, and the movement toward value‐based payments. This could include aligning climate goals for population health with goals for the built environment while maintaining a focus on health care prevention's triple aim: improving care, improving health, and lowering costs.

### 6.2 Emerging topics and research needs

Much is still unknown regarding climate change impacts on human health and safety generally and in New York State's diverse communities specifically. More research is needed across subdisciplines and subspecialties to complement the current state of knowledge. Examples of specific information gaps suggested by the literature or content experts involved in report development include:

- Regional variations in climate change impacts on health. New York could better characterize regional differences and then determine how regionally relevant approaches to improve outcomes related to climate change and health should proceed.
- Public health impacts of displacement due to sea level rise, including coping with the impending sense of loss. Lertzman refers to this phenomenon as “Environmental Melancholia.”^588^ Relatedly, the process of helping communities plan for managed retreat, specifically integrating their perspectives, is not yet well researched or fully understood in New York State or elsewhere in the United States.
- Robust, real‐time data on pollen levels. This data gap complicates epidemiologic investigation of current and potential future health burdens.
- Comprehensive data regarding locations of basement apartments in New York City and other areas of the state where such units are prevalent and at risk. This information is important for preventive flood warnings and safety measures during extreme precipitation and storm surge events.
- Regional costs of climate hazards that cause displacement and wildfire smoke, as these impacts are relevant to well‐being and public health. A 2018 study on regional costs of climate hazards for the buildings sector identified regional differences in expected climate impacts, but it did not include extreme heat (given that extreme heat has less direct impact on buildings), nor did it include sea level rise or wildfires.^589^
- Biological mechanisms that help account for the current epidemiologic evidence of heat wave exacerbation of mental health problems.^590^
- Impacts of reducing greenhouse gas emissions on Indigenous health.^591^
- The potential for cascading impacts and compounding events.

### 6.3 Conclusions

Human health and safety are fundamentally influenced by the environments in which people live, and climate change is radically altering these environments. New Yorkers are already experiencing escalating risks from the impacts of climate change. Nonclimate stressors compound climate challenges from heat, heavy rainfall, and flooding, posing threats to New Yorkers’ physical safety, physical health, mental health, and overall well‐being. The public and clinical health perspectives are critical considerations in planning initiatives to address these climate change threats. Health and safety can be further protected by an intentional approach that includes raising awareness, offering near‐term coping resources, developing hazard mitigation strategies that incorporate health outcomes, and framing adaptation strategies to include public health and health care system professionals.

This health and safety approach—as with all climate change strategy—risks perpetuating further harm on people of color, as well as Indigenous Peoples, unless it intentionally addresses the structural racism that amplifies climate change–related health risks for New Yorkers. In addition to specifically identifying these root causes of inequity, an additional important strategy is a “whole of community” approach that includes not only governance structures from local to state but also families, businesses, community‐based organizations, and institutions. To reduce disruption or suspension during increasingly frequent health crises, expanded climate change capacity at local health departments across New York State will need to include discrete leadership, funding, and staffing. While the health and safety threats from the changing climate loom large, the solutions could have substantial health benefits. The diverse solutions to protect health and safety across New York State through Climate Smart Communities and other initiatives demonstrate New Yorkers’ creativity in integrating climate change and health and, in turn, the potential for greater resilience.

## 7 TRACEABLE ACCOUNTS

Traceable accounts examine each key finding in depth. They provide citations that support each assertion and present the authors’ assessment of confidence in each finding.

### 7.1 Key Finding 1

**Climate change poses escalating health and safety risks for New Yorkers from heat, heavy rainfall, flooding, and air quality changes, combined with nonclimate stressors**. However, public health is not a key focus of most of the state's planning initiatives to address climate change. New York agencies and organizations can consider ways to reduce impacts by raising and tracking health risk awareness, offering near‐term coping resources, participating in the development of hazard mitigation strategies to incorporate health outcomes, and framing adaptation strategies to include training (for health professionals, the general public, and high‐risk populations) and health‐indicator monitoring.

#### 7.1.1 Description of evidence

National and New York State epidemiologic and clinical evidence supports the connection between climate hazards and human health risks, with the evidence base now consisting of hundreds of studies based primarily on public health surveillance data and medical records and complemented with climate‐health projection models.^17^, ^277^, ^403^ NYSDOH has been developing strategies in concert with a national learning community and is well‐positioned to provide input to other agencies on incorporating health protective approaches.^403^ Strategies to address these risks include outreach and education via public awareness campaigns, programs that offer short‐term strategies to different populations at risk of a variety of exposures, training programs for health professionals, and monitoring programs with indicators of health and degree of public concern.^403^, ^534^, ^592^, ^593^

#### 7.1.2 New information and remaining uncertainties

Localized climate‐health impact studies and robust evaluation of response strategies outside of large cities, particularly outside of New York City, are sparse.^134^, ^277^ Uncertainties exist around best practices for motivating behavior changes among individuals, institutions, and even municipal entities toward initiating climate actions that could reduce health risks and vulnerabilities.

#### 7.1.3 Assessment of confidence based on evidence

Given the evidence and remaining uncertainties, there is **high** confidence that without extensive intervention and reduction of vulnerabilities, New Yorkers’ health will be harmed by multiple climate hazards.

### 7.2 Key Finding 2

**Climate change–related impacts on mental health and well‐being are pronounced**. Heat, flooding, extreme storms, and other climate‐related events have documented, detrimental effects on mental health—especially for children and youth, older adults, people with pre‐existing mental health concerns, and those with limited access to mental health resources (such as many Indigenous and rural communities and unhoused individuals). Raising awareness of climate‐related mental health concerns can give New York health service providers the information they need to engage with patients on the topic and encourage actions to reduce exposure to climate hazards and their associated mental stresses, provide guidance on adaptations to deal with growing health risks, assuage anxieties, and offer grounded hope as well as applied solutions.

#### 7.2.1 Description of evidence

Research suggests that New Yorkers’ mental health and well‐being are affected by climate change through multiple mechanisms and over multiple time scales, and that these risks are increasing.^309^, ^312^, ^314^, ^316^ Extreme events and long‐term gradual changes in climate are linked to both mental health and well‐being risk.^311^ Increases in extreme heat, which are virtually certain across New York,^54^ suggest possible increases in violence and mental health problems.^320^, ^330^, ^331^, ^332^, ^333^ Risks are disproportionally shared across different demographic groups and other subpopulations.^239^, ^240^, ^241^, ^242^

New York can reduce impacts by prioritizing climate health risk awareness and preparedness—including for extreme weather events, heat‐related health impacts, and climate anxiety in general—using a “whole of community” approach.^36^, ^130^, ^313^, ^543^, ^594^, ^595^ This includes finding ways to reduce exposures, particularly given confidence in growing heat and other climate hazards and emerging data about their interrelationships with pre‐existing conditions.

#### 7.2.2 New information and remaining uncertainties

To understand the extent of mental health impacts from climate‐related issues and their compounding interactions (such as extreme heat exposure following flood‐related displacement), New York State needs further site‐specific research.^312^ There is a need for better geographic coverage of communities across the state, as well as a better understanding of mental health impacts on directly affected populations, including climate professionals and educators. Particular attention is needed for Indigenous and rural communities, Black and other communities of color, and persons with pre‐existing mental health conditions and social disadvantages. In addition to mental health and well‐being impacts research, there is an urgent need for applied intervention work linked to mental health and well‐being. For example, emerging research suggests that interventions such as urban greenspaces designed to cool environments are effective in reducing violence and improving overall well‐being,^272^, ^339^, ^596^ but more piloting and evaluation would offer more specific guidance on interventions.^597^ Further, a better understanding of which interventions are most effective could help alleviate complex and chronic traumatic experiences in light of the longevity and scope of climate change.

#### 7.2.3 Assessment of confidence based on evidence

With well‐documented connections between extreme weather, temperature, other climate hazards, and mental and behavioral health disorders,^17^, ^72^, ^466^, ^467^ there is **high** confidence that rising future temperatures will require investments in a variety of mental health services to support New Yorkers.^312^

### 7.3 Key Finding 3

**Discriminatory systems and policies amplify climate change–related health risks for New Yorkers**. Structural racism—a particularly entrenched form of discrimination—refers to the many ways in which this discrimination against people of color and Indigenous Peoples exists within systems and policies and how this affects access to and the distribution of resources. Such systemic forms of discrimination are embedded across housing, employment, health care, and other systems and result in people of color and low‐income and Indigenous Peoples having less adequate access to care, worse health outcomes, and greater climate exposures than other populations, making them more vulnerable to climate change impacts. Addressing climate change–related health impacts means also addressing systemic discriminatory policies and practices—particularly structural racism—and their long‐term negative health outcomes.

#### 7.3.1 Description of evidence

Over the last several decades, the public health and medical research communities have refined how racism is quantified and incorporated into epidemiologic models, and continue to do so.^598^ While initially focused on interpersonal racism,^599^ the field has shifted to focus more on the upstream, systemic forces of structural racism either implicitly or explicitly included in historic and current practices and policies.^410^ The climate crisis itself is driven by structural racism,^600^ but myriad specific health outcomes—ranging from cardiovascular morbidity in adults to adverse maternal and birth outcomes—and health disparities overall are also linked to these structural‐level drivers,^599^, ^601^, ^602^ which in turn amplify vulnerability to climate‐related risks.^393^, ^600^ The burdens of disease and poor health, and the benefits of well‐being and good health, are inequitably distributed.^47^, ^603^ Inequitable distribution is driven by social, environmental,^413^ economic, and structural factors that shape health and are themselves distributed unequally. People of color suffer more, including having worse health outcomes and greater climate‐related risks.^575^ Recognizing the impacts on people of color and identifying opportunities to directly address the impact of structural racism and climate change are critical components to addressing this root cause of current and growing climate‐health disparities across these communities.^35^, ^393^, ^410^

#### 7.3.2 New information and remaining uncertainties exposures

There is extensive new literature on redlining and higher temperatures in urban areas, tracking of higher heat‐related mortality impacts in communities of color, and evidence of low‐income families experiencing increased flood exposure.^604^, ^605^ However, fewer studies have focused on neighborhood‐level mitigation and adaptation planning for these communities. In New York State, most of the analyses have occurred in New York City. With few community‐level adaptation plans in place statewide, the predominance of disinvestment historically is a proxy for inequities in transition planning; however, this should be examined more systematically and directly.

#### 7.3.3 Assessment of confidence based on evidence

Given the evidence and remaining uncertainties, there is **high** confidence that race‐biased policies and practices have historically and presently compounded vulnerabilities to deleterious climate exposures.

### 7.4 Key Finding 4

**A collaborative, health‐centric, “whole of community” approach is essential for addressing the urgency and broad impacts of climate change**. Municipal and county health departments are at the front lines for climate response. However, because climate impacts vary greatly between and within communities, adaptation strategy development would also benefit from including families, businesses, community‐based organizations, and institutions who are affected. Efforts to engage local health departments in municipal and state planning for climate change have begun, and drawing diverse community members and organizations into the process can be a valuable next step.

#### 7.4.1 Description of evidence

Local governments at the municipal and county level are at the front lines for climate response and may share some of these responsibilities with other governmental units in New York State, such as local fire districts, school districts, and water districts. NYSDOH and NYSACHO held climate and health adaptation workshops for local health departments and their partners in the fall of 2022 and subsequently provided small technical assistance mini‐grants to 10 departments in the spring of 2023 to use toward climate and health priorities, making the best of limited resources.^606^ Following conversations with local health departments in New York State, NYSACHO concluded that “the impacts of climate change on the health of local communities are as varied as the place and the people.”^606^ A survey of New York State health department officials showed that the majority perceive climate change as relevant to public health and report some programmatic work that already uses or plans to use climate adaptation strategies, but nearly half did not know what, if any, resources would improve their department's ability to deal with climate change. Some health department officials perceived issues related to climate change as outside of their designated role, despite these issues having a clear link to public health.^32^ Within the last decade, federal agencies and climate‐focused nonprofits further emphasized the importance of “whole of community” approaches, offering guidance on multistakeholder engagement, cross‐sector collaborations, and iterative approaches to improve processes and outcomes.^36^, ^37^, ^545^, ^594^, ^595^

#### 7.4.2 New information and remaining uncertainties

To address these disconnects, New York State will need more integrated support for local health departments, much like the Health in All Policies commitment. New York issued Executive Order No. 190 regarding Health in All Policies, which “systematically considers the health implications of decisions made by all government entities regarding public policies; avoids harmful health impacts to improve population health and health equity; and incorporates health considerations into policies, programs, and initiatives led by non‐health agencies.”^607^ However, the challenge extends beyond state and municipal governance to include families, businesses, community‐based organizations, and institutions. While a New York State “whole of community” approach to public health and climate change is not underway, Executive Order No. 190 invites local health departments to take part in municipal and state planning for climate change. Drawing community members into the process is an important next step.^36^, ^594^, ^595^, ^608^

#### 7.4.3 Assessment of confidence based on evidence

Given the New York State survey results, there is **high** confidence that local health departments must be part of an overall health‐centered support network to address the myriad health‐related issues and interventions needed due to climate change.

### 7.5 Key Finding 5

**Public health efforts to address climate change must be sustained to be effective**. Public health agencies face competing challenges, and their climate‐related efforts are susceptible to suspension when other health crises occur. If New York wants to sustain public health agencies’ focus on climate change, local health departments will need discrete leadership, funding, and staffing. Given the projected rise in disease exposure and climate‐related increases in pandemics, this represents a critical step in preparing health systems, governments, and individuals.

#### 7.5.1 Description of evidence

As evidenced by NYSACHO's survey on Climate and Health Adaptation in New York State, additional resource commitment is needed to sustain public health agencies’ focus on climate change as disasters or other public health emergencies, such as the recent pandemic, divert resources from other public health services.^573^ During the recent pandemic response to COVID‐19, New York municipalities redirected climate‐focused health resources to COVID‐19 response, disrupting efforts intended to address climate change and health.^583^, ^606^ Although New York adopted a stringent Climate Act in 2019,^576^ local health departments struggled to tackle compounding events.^577^ For example, although New York City quickly rolled out a COVID‐19 Heat Wave Plan, shelters closed due to COVID‐19‐related limitations on gathering sizes.^582^, ^583^ Establishing programmatic strategies to enable interrelated challenges to coexist and retain necessary resources is a fundamental challenge for planning and budgeting purposes. Given the projected rise in disease exposure^565^, ^566^ and climate‐related increases in pandemics, this represents a critical step in the preparation of health systems of governments and individuals.

#### 7.5.2 New information and remaining uncertainties

COVID‐19 is not expected to be unique, but instead a continuation of increasing zoonoses. The type, pace, and extent of zoonoses remain uncertain, although expansion is certain.^22^, ^23^, ^24^, ^562^, ^609^, ^610^ A holistic approach to prepare public health agencies for multiple, compounding events is necessary to address the implications of another pandemic. Such an approach would recognize interdependencies (such as energy insecurity, extreme heat, and pandemic interactions), along with their complex and cascading interactions and scales (such as access to cooling at home during heat waves and access to cooling facilities due to pandemic restrictions). This preparation would allow for improved prediction of, preparation for, and adaptation to climate change.^24^, ^25^, ^611^ Additional research is required to understand the capacities of local health departments to focus on climate change preparedness in concert with pandemic preparedness and response, and to build such capacity if needed.

#### 7.5.3 Assessment of confidence based on evidence

Given evidence from COVID‐19 disruptions to planned health agency programs on climate change, there is **high** confidence that future health crises will have similar impacts.

## AUTHOR CONTRIBUTIONS

J.B.: Drafting, revising, and editing the manuscript; review and general supervision. P.S.: Drafting, revising, and editing the manuscript; review and general supervision. N.G.: Drafting, revising, and editing the manuscript. S.J.: Drafting, revising, and editing the manuscript. K.L.: Drafting, revising, and editing the manuscript. V.S.L: Drafting, revising, and editing the manuscript. F.M.: Drafting, revising, and editing the manuscript. A.S.: Drafting, revising, and editing the manuscript. M.S.: Drafting, revising, and editing the manuscript. S.S.: Drafting, revising, and editing the manuscript.

## COMPETING INTERESTS

The authors declare no competing interests.

### PEER REVIEW

The peer review history for this article is available at https://publons.com/publon/10.1111/nyas.15244.
